# Determinants of urban mosquito population density and community responses: A cross-sectional study

**DOI:** 10.12688/f1000research.164704.2

**Published:** 2025-09-01

**Authors:** Rekha T, Jithin Surendran, Sreedevi SR, Aishwariya Narasimhan, Nihal R Shankar, Arhan Vilas K, Gayana Shree A N, Hari Priya Muppala, Faiz Abdulaziz, Rohan HS, Ameya Singh

**Affiliations:** 1 Department of Community Medicine, Kasturba Medical College Mangalore, Manipal Academy of Higher Education, Manipal, India; 2 Kasturba Medical College Mangalore, Manipal Academy of Higher Education, Manipal, India

**Keywords:** Vector Borne Diseases, Malaria, Dengue, Epidemiologic Factors, Mosquito Control

## Abstract

**Background:**

Vector-borne diseases transmitted by various arthropods account for approximately 17% of the global burden of infectious diseases. These arthropods, especially mosquitoes, are particularly rampant in Mangalore because of the humid coastal climate and scaling urbanization.

**Objectives:**

To identify the key determinants of mosquito presence in urban settings and assess community-based prevention strategies and control measures. Also to evaluate community perceptions of mosquito-borne diseases and quantify their burden from self-reported cases.

**Methods:**

The study involved households in selected wards of the urban field practice area of the Department of Community Medicine, selected through convenience sampling. Data were collected using a semi-structured questionnaire covering sociodemographic details, mosquito proliferation, breeding determinants, behavioral measures, perception of mosquito control, and self-reported cases of mosquito-borne diseases. The data were analyzed using Jamovi version 2.6.26.

**Results:**

The study included 95 household participants, primarily female (70.5%) and literate individuals (94.8%). The 42.1% reported an increase in mosquito breeding sites over the past year and 69.4% recognized the rainy season, where mosquitoes were more prevalent. Seventy five percentage responded to water stagnation, which contributed to vector breeding. The survey showed, 91.5% of households used chemical measures as mosquito preventive measures. Ninety two percentage of respondents are aware of mosquito-borne diseases and 80% perceived regular environmental cleaning is a crucial method to prevent disease outbreaks. Thirty percentage of participants had suffered any mosquito-borne disease past year. Water stagnation (p = 0.033) and construction activity (p = 0.014) were significantly associated with a higher number of mosquitoes in the study setting.

**Conclusion:**

This study reveals a gap in community knowledge and perception of mosquito-borne diseases, even though people are aware of basic precautions, such as using mosquito sprays and screens. However, proper intervention by local authority is needed to combat breeding factors, such as water stagnation and dense vegetation.

## Introduction

Vectors are defined as organisms that transmit infectious pathogens from humans to humans, or from animals to humans. Some of the most prevalent vector-borne diseases include malaria, filariasis, dengue, chikungunya, yellow fever, chagas disease, bubonic plague, and leishmaniasis are transmitted by arthropod vectors. These diseases contribute 17% of infectious diseases that affect the human population worldwide and cause approximately 7,00,000 deaths per year. ^1^

Mosquitoes are one of the most prominent arthropod vectors, representing a significant portion of the vector-borne disease burden, with over 80% of the global population are at risk. ^1^ Mosquitoes are arthropods of medical importance under the class Insecta and are further divided into Anopheline and Culicine mosquitoes. Anopheline mosquitoes, which are responsible for malaria transmission, exhibit nocturnal biting habits (typically bites between 10 PM and 4 AM) and indoor resting behavior and breed in clean, sunlit water sources. India accounts for 79% of the global malaria burden, underscoring its public health significance. ^2– 4^

Culicine mosquitoes, notably *Aedes aegypti* and *Aedes albopictus*, are highly domesticated; they breed in water-filled containers in domestic and peridomestic areas and bite primarily during the day and evening. These species transmit dengue and chikungunya, with Asia accounting for 70% of the global dengue risk, and India being a significant contributor. ^5– 7^ The first chikungunya outbreak in India occurred in the 1960s, followed by a period of dormancy until a major resurgence in 2006, which affected 13 states in the country. ^8, 9^

India’s vulnerability to mosquito-borne diseases is exacerbated by its eco-socio-demographic conditions, making it a major public health concern. Karnataka, a southern state in India, faces a significant burden of mosquito-borne diseases including malaria, dengue, lymphatic filariasis, and Japanese encephalitis, with the prevalence varying across districts. In 2010, Karnataka reported 1,09,118 malaria cases, 28,065 of which were attributed to Plasmodium falciparum. ^10^

The coastal cities of Mangalore and Udupi together account for approximately 72% of the malaria cases reported in Karnataka. ^11^ In particular, Mangalore—a coastal town in the Dakshina Kannada district with frequent heavy rainfall and high humidity—provides an ideal environment for mosquito proliferation. An initial survey conducted in Mangalore taluk identified 26 mosquito species across six genera, with an annual parasite index of 10–12, indicating that the area was malaria endemic. ^12^ Rapid urbanization in urban Mangalore, including extensive construction, inadequate drainage, and poor road conditions, has further contributed to the persistence of the endemic nature of mosquito-borne diseases in the area. ^13^

Numerous studies have identified environmental, socioeconomic, and behavioral determinants of mosquito proliferation and vector-borne disease transmission. Aquatic habitats, such as pools, streams, and water-filled containers, are critical breeding sites for mosquitoes. For instance, a study by Wilke et al. (2020) in Miami-Dade Florida identified a few of the common aquatic habits that are responsible for harboring 80% of all immature *Ae. Aegypti* and the increase in proliferation in the presence of those aquatic habitats. ^14^ Another study by Prashanthi *et al.* (2007) showed that *Anopheles* breeds in pools and streams, where people living in close proximity are at high risk of malaria and its transmission. ^15^ These studies have also revealed that socioeconomic factors exacerbate the vulnerability to malaria, as economically marginalized populations often lack access to anti-mosquito measures, such as mosquito nets or repellents, and may follow age-old traditional practices, such as sleeping outdoors at night amid peak mosquito activity.

The use of preventive measures, such as effective lids over water storage containers and frequent emptying of containers, significantly reduces the incidence of arthropod proliferation, especially in *Ae. Aegypti.* ^16^ Studies have shown a linear relationship between growing populations, rising socioeconomic status, and increased mosquito proliferation, particularly in economically marginalized densely populated areas vulnerable to dengue. ^17^ A study by Srividya *et al.* (2018), through logistic regression analyses indicated that tiled and concrete dwellings increased the likelihood of an area becoming a dengue hotspot by 2.0 and 2.9 times, respectively, due to the conducive breeding environment. ^18^

There is a significant relationship between rapid, unplanned urbanization and the proliferation of mosquitoes. One study revealed that inadequate urban infrastructure and sanitation play important roles in the transmission and reproduction of vectors, especially *Aedes aegypti*. These unplanned disorganized cities aggravate mosquito proliferation by creating artificial breeding grounds, such as stagnant water pools, and increasing disease transmission. ^19^ Climate change also has a considerable impact on vector proliferation. It reduces larval development time and rapidly increases mosquito populations. This also leads to a reduction in the extrinsic incubation period of pathogens in mosquitoes, thereby increasing their infectiousness. ^19^

Community knowledge and behavior are critical for effective vector control. A study by Garbin et al. (2021) revealed that while 76% of respondents believed that their neighborhood was likely to be infected by a disease spread by mosquitoes, but no action was taken by them, highlighting a gap between awareness and actions. ^20^ Another study by Madeira et al. (2002) demonstrated that didactic interventions among schoolchildren increased knowledge about mosquito breeding sites and vector proliferation, leading to heightened awareness. ^21^

Various determinants of mosquito proliferation have been identified across different studies, and the present study builds on this evidence by exploring the specific environmental, socioeconomic, and behavioral factors driving mosquito proliferation in Mangalore and assessing community measures to mitigate vector-borne disease risk. By examining vector nuisance, disease prevalence, and community engagement, this study aligns with Sustainable Development Goal 3.3—ending epidemics of malaria and other communicable diseases. The results provide valuable evidence to support urban vector control programs and the development of tailored community-based interventions.

## Objectives

To identify the key determinants of mosquito presence in urban settings and assess community-measures taken to prevent an increase in mosquito density and control mosquito-borne diseases. Also to evaluate the community perceptions towards mosquito-borne diseases and quantify the disease burden from self-reported cases.

## Methodology

This community-based cross-sectional study was conducted in Mangalore, a coastal city in the South Indian state of Karnataka, between September and October 2024, among Community households present in the urban field practice area of the Department of Community Medicine. The sample size was calculated based on a previous study conducted in Mangalore, Karnataka, ^22^ it was reported that 83% of the people used preventive measures such as mosquito nets to prevent mosquito bite, using this as our anticipated proportion and 10% relative precision, 95% confidence interval, and adding 20% non-response rate as 95 sample size was calculated as follows:

N = Z α 2pq/d2: Z = 1.96, 95% confidence interval; p = 0.83; q = 1-p = 0.17, and d is 10% relative precision and 20% of non-response rate.

The study protocol was approved by the Institutional Ethics Committee, Kasturba Medical College Mangalore with No: IEC KMC MLR 09/2024/587, followed by permission from the Head of the Institute. The written consent was taken on an ‘Informed consent form’ which was provided to the participants ≥18 years of age for signature, and the participants <18 years of age were excluded from the study. All participants had the right to withdraw at any stage of the study, and all incomplete responses were considered withdrawal and excluded from the analysis. The study participants were approached from a household that was selected from five wards out of the total 60 wards present under the Mangalore City Corporation based on convenience; one reliable informant residing in the household for more than at least 1 year, who was aware of the household conditions and consented were included in the study, whereas temporary residents of less than a year, apartment complexes, and households without adult personnel were excluded.

The number of households to be taken for our study was divided equally among each ward; that is, a total of 19 households from each of the selected wards were considered for the study. A random street was selected from each ward and then, standing at the end of each randomly selected street, every 3 ^rd^ house present on the left side of the lane was considered for the study, which was continued in a clockwise order; in case of failure to meet the inclusion criteria, absence, or denial to take part in the study, the next house was considered. When the head of the household was absent during the time of data collection, information was collected from the oldest adult residing in the household. A pretested, semi-structured, internally validated physical questionnaire designed after extensive literature review was used for data collection, which comprised details such as sociodemographic data, mosquito proliferation and nuisance, determinants of mosquito breeding, behavioral and preventive measures, perception related to mosquito control, and self-reported cases of mosquito-borne diseases. Data were recorded after obtaining informed consent from the heads of the households.

The data collected were entered into MS Excel and analyzed using Jamovi version 2.6.26. Descriptive statistics are represented using frequencies and proportions. The association between two categorical variables was assessed using the chi-square test. The strength of association between the independent and dependent variables were measured using odds ratios (OR) with corresponding 95% confidence intervals (CI) and significant *p* value of <0.05. Multivariate logistic regression was performed to assess the independent effect of overlapping risk factors.

## Results

**Table 1.** Socio-demographic characteristics of study participants (N = 95).

Socio-demographic details	n (%)
**Age (in years)**	
18-30	12 (12.6)
31-45	14 (14.7)
46-60	34 (35.8)
>60	35 (36.8)
**Gender**	
Male	28 (29.5)
Female	67 (70.5)
**Education**	
Illiterate	5 (5.2)
Primary School	11 (11.6)
High School + PUC	46 (48.4)
Degree	33 (34.7)
**Proximity of Nearby Health Care facility (in km)**	
0-2	58 (61.1)
>2-4	25 (26.3)
>4	12 (12.6)

The study included 95 household, with majority of the participants were above the age of 45 years (72.6%), and most of them were females. 94.8% of them were educated and had access to nearby healthcare services <2 km ( Table 1).

**Table 2.** Perceived mosquito proliferation and nuisance (N = 95).

Perception	n (%)
**Increase in mosquito breeding sites in the last 1 year (yes)**	40 (42.1)
**Presence of mosquitoes inside the house**	
Mild	31 (32.6)
Moderate to Severe	64 (67.4)
**Presence of mosquitoes outside the house**	
Mild	12 (12.6)
Moderate to Severe	83 (87.4)
**Time of the day when mosquitoes mostly bite**	
Morning	8 (8.4)
Evening	68 (71.6)
Night	19 (20.0)
**Mosquito bites causing disturbed sleep or discomfort at night**	29 (30.5)
**Weather conditions in which mosquitoes are mostly prevalent**	
Rainy season	66 (69.5)
Hot weather	16 (16.8)
No noticeable difference	13 (13.7)
**Overall mosquito nuisance in locality compared to past one year**	
Mild	38 (40.0)
Moderate to Severe	57 (60.0)

Among the study participants, 42% of them noticed an increase in mosquito breeding sites last year, along with an increase in the presence of mosquitoes both inside and outside the house. They also noticed a widespread nuisance of mosquitoes during the evening, which was more prevalent during the rainy season ( Table 2).

**Table 3.** Determinants of mosquito breeding (N = 95).

Determinants of mosquito breeding *	n (%)
**Water stagnation (n = 71)**	71 (74.7)
Puddles	53 (74.6)
Flowerpots	60 (84.5)
Construction sites	23 (32.3)
**Garbage Dumping**	13 (13.6)
**Presence of water body**	19 (20.0)
**Presence of dense vegetation**	52 (54.7)
**Water storage in uncovered containers**	12 (12.6)
**Presence of water leaks or overflow from pipes and tanks**	8 (8.4)

Most common determinants of mosquito breeding noticed by the participants were water stagnation and presence of dense vegetation, which were 74.7% and 54.7%, respectively ( Table 3).

* Multiple responses.

**Table 4.** Behavioural and preventive measures by participants for mosquito control (N = 95).

Preventive measures		n (%)
**Type of measure** *		
Chemical		87 (91.5)
Mosquito nets, window screens or meshes		53 (55.7)
Closing doors and windows		77 (81.0)
Other personal measures		15 (15.7)
**Mosquito repellent/coils**		
Yes, daily		24 (25.2)
Yes, occasionally		23 (24.2)
Never		48 (50.5)
**Insect repellent before sleep**		
Yes		12 (12.6)
No		83 (87.3)
**Cleaning of surroundings**	**By household members**	
	Daily	49 (51.5)
	Weekly	31 (32.6)
	Occasionally	15 (15.8)
	**By municipality**	
	Yes	39 (41.0)
	No	56 (58.9)
**Water stagnation (Checking and eliminating stagnation)**		
Daily		17 (17.8)
Weekly		37 (38.9)
Occasionally		30 (31.5)
Never		11 (11.5)
**Check holes in window screens/mosquito nets**		
Regularly (monthly/more)		24 (25.2)
Occasionally		17 (17.8)
Rarely		17 (17.8)
Never		37 (38.9)
**Anti-mosquito fogging by local authorities**		
Frequently #		6 (6.3)
Occasionally		41 (43.1)
Never		48 (50.5)

Most participants used insecticide coils/vaporizers, closed doors and windows, and had window screens as personal preventive measures against mosquitoes. The majority of them regularly clean their surroundings and check for water stagnation around their houses ( Table 4).

* Multiple responses.

^#^ Frequently: weekly or monthly.

**Table 5.** Awareness and perception of mosquito borne diseases and mosquito control measures (N = 95).

Awareness and perception	n (%)
**Awareness about mosquito borne disease** *	88 (92.6)
Malaria	85 (96.6)
Dengue	82 (93.2)
Chikungunya	29 (33)
Zika	8 (9.1)
Filariasis	9 (10.2)
**Perception of Preventive measures to be taken to avoid Mosquito-borne diseases** *	
Regular cleaning of surroundings	76 (80.0)
Eliminating stagnant water	71 (74.7)
Usage of insecticide sprays	50 (52.6)
Usage of mosquito nets	46 (48.4)
Education and spreading awareness	92 (96.8)
**Perception of community on determinants of mosquito proliferation**	
**a) Water stagnation**	
Clean water	16 (16.8)
Dirty water	44 (46.3)
Both clean and dirty water	35 (36.8)
**b) Seasonal variation**	85 (89.4)
Rainy season	69 (81.1)
Summer season (Dry Hot weather)	16 (18.8)

A majority of 90% of the participants were aware of different mosquito borne diseases and the various preventive measures to be taken for its prevention. They were also adequately perceived as determinants of mosquito proliferation, such as water and seasonal variation ( Table 5).

* Multiple responses.

**Table 6.** Self-Reported cases and outcome of mosquito borne diseases (N=95).

Self-Reported cases	N (%)
**Suffered from any mosquito-borne disease in the past 1 Year**	
Yes	28 (29.4)
**Mosquito borne Disease (N = 28)**	
Malaria	8 (28.5)
Dengue	20 (71.4)
**Symptoms** *	
Fever	27 (96.4)
Headache	22 (78.5)
Joint Pain	17 (60.7)
Rash	2 (7.1)
Muscle Pain	13 (46.4)
Others	17 (60.7)
**Place of Treatment**	
Public Health Centre	5 (17.8)
Private Clinic	23 (82.1)
**Complications after recovery** *	
Weaknesses	12 (92.3)
Cold	1 (7.6)
Leg Pain	1 (7.6)
Headache	1 (7.6)
Eye Pain	1 (7.6)

Approximately 30% of the participants reported suffering from a mosquito borne disease in the last one year. Most patients have symptoms such as fever, headache, joint pain, and muscle pain. Most of them (82%) sought treatment from private clinics, within one week of appearance of symptoms and recovered within 2 weeks ( Table 6).

* Multiple choice question.

**Table 7.** Association of environmental factors with mosquito density: univariate and multivariate logistic regression analysis (N=95).

Variable	Increased mosquito density		Unadjusted OR (95% CI)	p value	Adjusted OR (95% CI)	p value
	Yes (%)	No (%)				
	N = 49	N = 46				
**Presence of water stagnation** Yes No	34 (69.3) 15 (30.6)	22 (47.8) 24 (52.1)	**2.5 (1.1, 5.7)**	0.033 *	2.1 (0.9, 5)	0.097
**Presence of construction sites** Yes No	17 (34.6) 32 (65.3)	6 (13.0) 40 (86.9)	**3.5 (1.2, 10.0)**	0.014 *	**3 (1, 8.8)**	0.042 *
**Presence of any water body (pond, lake, etc.)** Yes No	8 (16.3) 41 (83.6)	7 (15.2) 39 (84.7)	1.1 (0.4, 3.3)	0.882	-	-
**Presence of Dense Vegetation** Yes No	31 (63.2) 18 (36.7)	21 (45.6) 25 (54.3)	2.1 (0.9, 4.6)	0.085	-	-
**Presence of Garbage dumping sites** Yes No	8 (16.3) 41 (83.6)	5 (10.8) 41 (89.1)	1.6 (0.5, 5.3)	0.439	-	-
**Type of Water storage** Covered containers Uncovered containers	41 (83.6) 8 (16.3)	42 (91.3) 4 (8.6)	2.1 (0.6, 7.3)	0.263	-	-
**Presence of water leaks or overflow from tanks or taps** Yes No	6 (12.2) 43 (87.7)	2 (4.3) 44 (95.6)	3.1 (0.6, 16.1)	0.166	-	-

The presence of water stagnation (OR 2.5, 95% CI: 1.1, 5.7) and construction sites (OR 3.5, 95% CI: 1.2,10.0) were significantly associated with increased mosquito density. However, after adjusted for water stagnation, only the construction sites (OR 3.0, 95% CI: 1.0, 8.8) remained significantly associated with mosquito density. This indicates that areas without construction sites had considerably lower mosquito density compared to areas with construction activity ( Table 7).

OR = Odds Ratio; CI = Confidence Interval.

* Statistically significant (p value ≤ 0.05; χ ^2^ test).

**Table 8.** Association between mosquito borne disease prevalence and Preventive measures taken inside the house (N = 95).

Variable	Presence of mosquito-borne disease		OR (95 CI%)	p Value
	Yes (%)	No (%)		
	N = 28	N = 67		
**Repair or check holes in window screens or mosquito nets** Yes No	9 (32.1) 19 (67.8)	32 (47.7) 35 (52.2)	0.5 (0.2, 1.2)	0.161
**Use of mosquito repellents or coils** Yes No	20 (71.4) 8 (28.5)	27 (40.3) 48 (59.7)	**3.7 (1.4, 9.6)**	0.006 *
**Use of electric mosquito bats or insecticide sprays** Often Rarely	11 (39.2) 17 (60.7)	22 (32.8) 45 (67.1)	1.3 (0.5, 3.3)	0.547
**Usage of Mosquito nets at night** Yes No	14 (50.0) 14 (50.0)	37 (55.2) 30 (44.7)	0.8 (0.3, 1.9)	0.642

The use of mosquito repellents or coils was significantly associated with presence of mosquito borne diseases (OR 3.7, 95% CI: 1.4, 9.6), which may reflect reverse causation, whereby individuals in high mosquito-density areas are more likely to use these preventive measures ( Table 8).

OR = Odds Ratio; CI = Confidence Interval.

* Statistically significant (p value ≤ 0.05; χ ^2^ test).

## Discussion

In the present study, a majority (95%) had some level of education, and 61% reported nearby healthcare facilities within 2 km, indicating relatively good access to healthcare in the study area. These findings match those of prior studies highlighting that educational level and proximity to health services may influence awareness and preventive behaviors related to vector-borne diseases. ^20, 21^ Approximately 42% of participants reported an increase in mosquito breeding sites in the past year, and 60% of participants reported experiencing moderate to severe mosquito nuisance in their locality over the past year. This contrasts with the findings of Davidson et al., who noted that 80% of respondents in St. Johns County, Florida, felt bothered by mosquitoes daily or several times a week. ^23^ Notably, 71.5% of the participants indicated that evenings were the peak time for mosquito activity, and 30.5% reported that mosquito bites disrupted their sleep, which is consistent with *Aedes aegypti’s* day-biting habits, whereas Anopheline mosquitoes are known for nocturnal activity. ^15^

Additionally, 67.4% of the participants described a moderate to severe presence of mosquitoes indoors, while 87.3% reported similar conditions outdoors. This finding is comparable to data from Kampango et al., who reported an average of 85.93% An. gambiae s.l. bites per night, with 66% occurring indoors and 34% outdoors, peaking between 22:00 and 03:00 in a rural community in Chókwè District, southern Mozambique. ^24^

In the present study, 50% of the mosquitoes were often found inside the house, whereas 48.3% of the population rarely found mosquitoes inside the house after the use of mosquito repellents or coils. A total of 64.5% of the population rarely found mosquitoes inside houses, whereas 32.8% often found mosquitoes inside houses after regular repair or checking holes in window screens or mosquito nets. A total of 61.2% of the population rarely found mosquitoes inside houses without usage of insecticide sprays at home. A total of 84.3% often found mosquitoes inside the house when they stored water in closed containers. Those who often repaired or checked holes in window screens and mosquito nets were more likely to report a lower presence of mosquitoes than those who rarely performed the repair.

Considering the environmental factors, 69.4% of the participants identified the rainy season as the period when mosquitoes were most prevalent. This aligns with a cross-sectional study by Mahgoub et al. in Barakat and El-Kareiba, Sudan, which reported a high number of positive habitats during the rainy season, whereas the lowest numbers were reported during the hot season followed by the dry season, corroborating findings that climate and seasonal variation significantly influence mosquito density and disease transmission. ^19, 25^

The primary breeding sites identified in our study included water stagnation (74.7%), dense vegetation (54.7%), and nearby water bodies (20%), which is consistent with previous studies demonstrating the importance of stagnant water and vegetation in vector proliferation. ^14^^–^^16^ In contrast, Mahgoub et al. reported that the main breeding sites for various mosquitoes in Sudan were leaking water pipes (51.5%), followed by irrigation channels (34.2%), hoof prints (6.4%), tire tracks (5.5%), and water tanks (2.4%). ^25^ Logistic regression analysis in this study revealed that water stagnation (OR 2.5, 95% CI: 1.1, 5.7 p value = 0.033) was significantly associated with a greater presence of mosquitoes, suggesting that stagnant water plays a crucial role in mosquito proliferation, and the absence of construction (OR 3.0, 95% CI: 1.0, 8.8 p value = 0.014) activity was significantly associated with a lower presence of mosquitoes. Multivariate logistic regression analysis, adjusting for water stagnation, showed that only the presence of construction sites remained significantly associated with increased mosquito density (OR 3.0, 95% CI: 1.0–8.8 p value = 0.042). These findings suggests that construction sites may serve as important breeding grounds for mosquitoes and that areas without construction activity experience considerably lower mosquito density. These findings highlight the need for targeted vector control measures at construction sites to reduce mosquito proliferation. ^18, 19^

Preventive measures among participants were notable, as 91.5% used chemical repellents (insect sprays, vaporizers, and smoke), 81% kept doors and windows closed, 55.7% utilized mosquito nets or screens, and 15.7% employed other personal measures. This contrasts with a study in urban northern Gujarat by Mahalakshmi et al., in which 67.3% used chemical measures such as repellent creams or liquids, 22% used bed nets, and only 2% reported taking no precautionary measures. ^26^

In terms of community cleaning practices, 51.5% of individuals reported cleaning their surroundings daily; additionally, 38.9% checked for water stagnation weekly; in contrast, 84.6% never reported water stagnation to authorities. Unfortunately, 58.9% indicated that municipal cleaning was infrequent in the locality. Notably, 25.2% of the participants regularly checked window screens or mosquito nets for damage, and 50.5% stated that there had been no anti-fogging efforts by local authorities. This is compared with Mahalakshmi et al.’s findings in northern Gujarat, where 88% cleaned their homes daily, 57.3% cleaned their surroundings weekly, and 82% actively avoided water stagnation. ^26^

Despite these measures, approximately 30% of the participants reported suffering from a mosquito-borne disease in the past year, with dengue accounting for 71.4% of the cases and malaria accounting for (28.6%). Common symptoms included fever (96.4%), headache (78.5%), joint pain (60.7%), and muscle pain (46.4%), and 82% of the patients sought treatment at private clinics. This aligns with findings from a study conducted by Kumar et al. in a tertiary hospital in Udupi district, which indicated that 83.9% of cases were due to dengue fever, presenting symptoms such as fever (99.1%), myalgia (64.6%), vomiting (47.6%), headache (47.6%), abdominal pain (37.5%), and breathlessness (17.8%). ^27^

Finally, 92.3% of the participants in our study reported experiencing complications postrecovery, primarily weakness, whereas 7.6% experienced cold, leg pain, headache, or eye pain. This contrasts with a study in Vietnam by Tam et al., who reported that 12.5% of participants experienced alopecia, 11.1% had blurred vision, 9.5% faced concentration difficulties, and 8.5% suffered from fatigue following dengue infection. ^28^

The present study found that 82.1% of the population perceives water stagnation as a significant factor in mosquito proliferation, indicating that it is a major factor in the prevalence of mosquito-borne diseases. Construction sites, water bodies, dense vegetation, and water storage in open containers were identified as significant determinants. Additionally, 17.8% of individuals perceive water leakage from tanks and taps (p = 0.166) as a key determinant, indicating that water leakage from household stores is a significant factor in the prevalence of mosquito-borne diseases. Overall, these factors contribute to the prevalence of mosquito-borne diseases.

Interestingly, the use of mosquito repellents or coils was significantly associated with a higher incidence of mosquito-borne diseases (OR 3.7, 95% CI: 1.4, 9.6, p = 0.006). This odd finding may indicate that individuals resort to chemical measures instinctively in areas with high mosquito density rather than those measures that effectively reduce disease risk. This finding also suggests possible improper usage or overreliance on repellents without addressing environmental breeding sites. Similar gaps between knowledge and action have been reported in prior studies. ^20^

## Conclusion

In conclusion, the study adopts a novel, integrated approach by identifying key determinants of mosquito presence while simultaneously evaluating community measures, perception and self-reported disease burden, provide context specific insights from Mangalore that extend beyond previous studies in other settings. The study showed the knowledge and attitudes regarding mosquito borne diseases in the community are apt and good. It correlates with good practice but only to a certain extent. Practicing the basic requirements, for example, using mosquito sprays and closing windows, is to par. However, proper intervention by local authority is necessary to combat the main factors responsible for mosquito breeding, such as water stagnation and the presence of dense vegetation. This proves the presence of gaps identified by our study, where despite most people being aware of mosquito-borne diseases, respondents recognized the lack of awareness and good preventive practices. Hence, apart from awareness, there is a dire need to provide the right personnel and services to combat mosquito-borne diseases.

## Recommendation

### Awareness and campaigns

Nearly hundred percent of participants believe that spreading awareness about mosquito-borne diseases and mosquito control measures will help in reducing mosquito borne diseases in the community. This can be accomplished through various means, such as public service announcements, social media campaigns, and community outreach programs.

### Elimination of water stagnation

Local authorities and communities must proactively repair damaged roads, cover open drains, strengthen vector control at construction sites, and ensure proper waste disposal to eliminate standing water, prevent mosquito breeding, and reduce the risk of vector-borne diseases.

### Clearing up of dense vegetation

The presence of dense vegetation was a significant determinant of the presence of mosquitoes in household neighborhoods. Local authorities should ensure regular clearing of vegetation and maintenance of cleanliness in these areas.

### Further research

Further research to identify the factors leading to an increase in the mosquito population and community and local government measures can help recognize additional intervention strategies. This can involve qualitative research methods, such as in-depth interviews, field research, or focus groups, to gain a deeper understanding of the underlying issue.

## Ethical approval

The study protocol was approved by the Institutional Ethics Committee, Kasturba Medical College Mangalore with No: IEC KMC MLR 09/2024/587, followed by permission from the Head of the Institute. The written consent was taken on an ‘Informed consent form’ which was provided to the participants ≥18 years of age for signature, and the participants <18 years of age were excluded from the study. All participants had the right to withdraw at any stage of the study, and all incomplete responses were considered withdrawal and excluded from the analysis.

## Data Availability

The data set used and/or analyzed during the current study are available from the online repository (figshare) DOI- https://doi.org/10.6084/m9.figshare.29144975. ^29^ ( Tables 1– 8) The data include participant information sheet, Informed consent form and questionnaire are available from the online repository (figshare) DOI- https://doi.org/10.6084/m9.figshare.29144990. ^30^ Data are available under the terms of the Creative Commons Zero “No rights reserved” data waiver (CC0 Public domain dedication).

## References

[1] WHO: *Global vector control response 2017–2030.* WHO;2017.

[2] Huho B Briët O Seyoum A : Consistently high estimates for the proportion of human exposure to malaria vector populations occurring indoors in rural Africa. *Int. J. Epidemiol.* 2013;42(1):235–247. 10.1093/ije/dys214 23396849 PMC3600624

[3] Pluess B Tanser FC Lengeler C : Indoor residual spraying for preventing malaria. *Cochrane Database Syst. Rev.* 2010;2010(4). 10.1002/14651858.CD006657.pub2 PMC6532743 20393950

[4] World Health Organization: *World malaria report 2022.* Geneva: World Health Organization;2022. Reference Source

[5] National Center for Vector Borne Disease Control: *Operational guidelines for hospitals to control Aedes breeding.* New Delhi: National Center for Vector Borne Diseases Control;2022. Reference Source

[6] Bhatt S Gething PW Brady OJ : The global distribution and burden of dengue. *Nature.* 2013;496(7446):504–507. 10.1038/nature12060 23563266 PMC3651993

[7] Brady OJ Gething PW Bhatt S : Refining the global spatial limits of dengue virus transmission by evidence-based consensus. *PLoS Negl. Trop. Dis.* 2012;6(8):e1760. 10.1371/journal.pntd.0001760 22880140 PMC3413714

[8] Paulson W Kodali NK Balasubramani K : Social and housing indicators of dengue and chikungunya in Indian adults aged 45 and above: Analysis of a nationally representative survey (2017-18). *Arch. Public Health.* 2022;80:125. 10.1186/s13690-022-00868-5 35443704 PMC9022351

[9] Chavasse D : Know your enemy: Some facts about the natural history of Malawi’s Anopheles mosquitoes and implications for malaria control. *Malawi Med. J.* 2002 Apr 1;14(1):7–8. 27528915 PMC3345420

[10] Kumar KR Gururaj G : Community Perception Regarding Mosquito-borne Diseases in Karnataka State, India.

[11] Prasad KI Govindarajan R Sreepada KS : Seasonal Diversity of mosquito species in Dakshina Kannada district, Karnataka, India. *Journal of Vector Borne Diseases.* 2021 Apr 1;58(2):119–125. 10.4103/0972-9062.321758 35074945

[12] Dayanand KK Punnath K Chandrashekar V : Malaria prevalence in Mangaluru city area in the southwestern coastal region of India. *Malar. J.* 2017 Dec;16:1. 10.1186/s12936-017-2141-0 29258505 PMC5735873

[13] Ghosh SK Manju R : Malaria elimination in India—The way forward. *J. Vector Borne Dis.* 2019;56(1):32–40. 10.4103/0972-9062.257771 31070163

[14] Wilke AB Vasquez C Carvajal A : Proliferation of Aedes aegypti in urban environments mediated by the availability of key aquatic habitats. *Sci. Rep.* 2020 Jul 31;10(1):12925. 32737356 10.1038/s41598-020-69759-5 PMC7395141

[15] Prashanthi D Ranganathan CR Balasubramanian S : Socio-demographic determinants of Malaria in highly infected rural areas: regional influential assessment using GIS. GIS for Health and the Environment: Development in the Asia-Pacific Region With 110 Figures. 2007;195–205.

[16] Yadav K Dhiman S Rabha B : Socio-economic determinants for malaria transmission risk in an endemic primary health centre in Assam, India. *Infect. Dis. Poverty.* 2014 Dec;3:1–8. 10.1186/2049-9957-3-19 24991410 PMC4078389

[17] Telle O Vaguet A Yadav NK : The spread of dengue in an endemic urban milieu–the case of Delhi, India. *PloS one.* 2016 Jan 25;11(1):e0146539. 10.1371/journal.pone.0146539 26808518 PMC4726601

[18] Srividya A Subramanian S Sadanandane C : Determinants of transmission hotspots and filarial infection in households after eight rounds of mass drug administration in India. *Trop. Med. Int. Health.* 2018 Nov;23(11):1251–1258. 10.1111/tmi.13143 30152049

[19] Almeida LS Cota AL Rodrigues DF : Sanitation, Arboviruses, and Environmental Determinants of Disease: impacts on urban health. *Ciencia & saude coletiva.* 2020 Sep 28;25:3857–3868. 10.1590/1413-812320202510.30712018 32997018

[20] Garbin CA Teruel GP Saliba TA : Knowledge and attitude of women with high-risk pregnancy about the Zika virus transmission. *Ciênc. Saúde Colet.* 2021 Jan 25;26:233–240. 10.1590/1413-81232020261.25752018 33533844

[21] Madeira NG Macharelli CA Pedras JF : Education in primary school as a strategy to control dengue. *Rev. Soc. Bras. Med. Trop.* 2002;35:221–226. 10.1590/S0037-86822002000300004 12045814

[22] Maskeri R Jain A Ullal S : Knowledge, Attitude and Practices (KAP) Regarding Malaria and its Prevention among Patients with Suspected Malaria in Mangaluru. *Indian J. Public Health Res. Dev.* 2018 Sep 1;9(9):271–276. 10.5958/0976-5506.2018.01008.2

[23] Davidson CA Scott JM Hossain TH : Relationship between citizen knowledge, vegetation coverage, and frequency of requests for mosquito control service in St. Johns county, Florida. *Technical Bulletin of the Florida Mosquito Control Association.* 2016;10:30–38.

[24] Kampango A Pinto J Abílio AP : Characterisation of human exposure to nocturnal biting by malaria and arbovirus vectors in a rural community in Chókwè district, southern Mozambique. *Wellcome Open Research.* 2023;8:193. 10.12688/wellcomeopenres.19278.1 37484481 PMC10357080

[25] Mahgoub MM Kweka EJ Himeidan YE : Characterisation of larval habitats, species composition and factors associated with the seasonal abundance of mosquito fauna in Gezira, Sudan. *Infect. Dis. Poverty.* 2017 Dec;6:1. 10.1186/s40249-017-0242-1 28173839 PMC5297020

[26] Mahalakshmi B Sivasubramanian N Vaghela P : Awareness on mosquito borne diseases among urban & rural population in Northern Gujarat. *Bioinformation.* 2022;18(7):640–644. 10.6026/97320630018640 37313054 PMC10259226

[27] Kumar A Rao CR Pandit V : Clinical manifestations and trend of dengue cases admitted in a tertiary care hospital, Udupi district, Karnataka. *Indian J. Community Med.* 2010 Jul 1;35(3):386–390. 10.4103/0970-0218.69253 21031102 PMC2963875

[28] Tam DT Clapham H Giger E : Burden of postinfectious symptoms after acute dengue, Vietnam. *Emerg. Infect. Dis.* 2023 Jan;29(1):160–163. 10.3201/eid2901.220838 36573590 PMC9796196

[29] Surendran J Dr : VBD F1000Research data sheet.Dataset. *figshare.* 2025. 10.6084/m9.figshare.29144975

[30] Surendran J Dr : EXTENDED DATA VBD F1000RESEARCH.Dataset. *figshare.* 2025. 10.6084/m9.figshare.29144990

