# Determinants of urban mosquito population density and community responses: A cross-sectional study

**DOI:** 10.12688/f1000research.164704.3

**Published:** 2025-11-26

**Authors:** Rekha T, Jithin Surendran, Sreedevi SR, Aishwariya Narasimhan, Nihal R Shankar, Arhan Vilas K, Gayana Shree A N, Hari Priya Muppala, Faiz Abdulaziz, Rohan HS, Ameya Singh

**Affiliations:** 1 Department of Community Medicine, Kasturba Medical College Mangalore, Manipal Academy of Higher Education, Manipal, India; 2 Kasturba Medical College Mangalore, Manipal Academy of Higher Education, Manipal, India

**Keywords:** Vector Borne Diseases, Malaria, Dengue, Epidemiologic Factors, Mosquito Control

## Abstract

**Background:**

Vector-borne diseases transmitted by various arthropods account for approximately 17% of the global burden of infectious diseases. These arthropods, especially mosquitoes, are particularly rampant in Mangalore because of the humid coastal climate and scaling urbanization.

**Objectives:**

To identify key environmental and behavioral determinants of mosquito presence in urban Mangalore, to assess community-based prevention and control measures, and to evaluate community perceptions and self-reported disease burden.

**Methods:**

The study involved households in selected wards of the urban field practice area of the Department of Community Medicine, a teaching and service field area under the Mangalore City Corporation, selected through convenience sampling. Data were collected using a semi-structured questionnaire covering sociodemographic details, mosquito proliferation, breeding determinants, behavioral measures, perception of mosquito control, and self-reported cases of mosquito-borne diseases. The data were analyzed using Jamovi version 2.6.26.

**Results:**

Among 95 respondents (70.5% female and 94.8% literate), 42.1% reported an increase in mosquito breeding sites over the past year, 69.4% recognized the rainy season as the peak period of mosquito activity. Water stagnation [74.7% (95% CI: 64.8–83.1)] and ongoing construction activity [32.4% (95% CI: 21.8–44.1)] emerged as significant environmental determinants of higher mosquito density. A large majority of households (91.6%) reported using chemical measures for mosquito prevention, while 92.6% of participants were aware of mosquito-borne diseases and their modes of transmission. Despite this, nearly one-third (29.4%) of respondents had experienced a mosquito-borne illness in the preceding year, with 71.4% dengue infection. The use of mosquito repellents was paradoxically associated with a higher prevalence of mosquito-borne diseases (OR = 3.7; 95% CI: 1.4–9.6; p = 0.024).

**Conclusion:**

Although awareness and preventive measure uptake were high, gaps remain in consistent environmental control and municipal interventions. Strengthening local authority action on water stagnation and construction-site management is essential for sustainable vector control.

## Introduction

Vectors are defined as organisms that transmit infectious pathogens from humans to humans or from animals to humans. Common vector-borne diseases, including malaria, lymphatic filariasis, dengue, chikungunya, West Nile fever, yellow fever, Chagas disease, bubonic plague, and leishmaniasis, are transmitted by arthropod vectors. ^1^ Together, these diseases account for approximately 17% of all infectious diseases worldwide and are responsible for nearly 700,000 deaths annually. ^2^

Mosquitoes are among the most prominent arthropod vectors, representing a significant portion of the vector-borne disease burden, with over 80% of the global population at risk. ^1^ Mosquitoes are arthropods of medical importance under the class *Insecta* and are further divided into *Anopheline* and *Culicine* mosquitoes. *Anopheline* mosquitoes are the primary vectors of malaria; they generally exhibit nocturnal biting habits (typically bites between 10 PM and 4 AM) and indoor resting behavior and breed in clean, sunlit water sources. According to the World Health Organization (WHO), malaria alone accounted for approximately 249 million cases globally in 2023, with 94% occurring in the WHO African Region. ^3^ India contributes nearly 52% of malaria cases outside sub-Saharan Africa and represents approximately 79% of the malaria burden within the WHO Southeast Asia Region. ^4, 5^

Culicine mosquitoes, notably *Aedes aegypti* and *Aedes albopictus*, are highly domesticated; they breed in water-filled containers in domestic and peridomestic areas and bite primarily during the day and early evening. These species transmit viral infections such as dengue, chikungunya, and Zika, which contribute to tens of thousands of deaths per year despite causing hundreds of millions of infections. ^6– 8^ Recent WHO global dengue surveillance, from January to November 2024, the total number of dengue cases was 13,860,025, with a total of 9990 deaths. ^9, 10^ The first chikungunya outbreak in India occurred in the 1960s, followed by a period of dormancy until a major resurgence in 2006, which affected 13 states in the country. ^11, 12^

India’s vulnerability to mosquito-borne diseases is exacerbated by its eco-socio-demographic conditions, making it a major public health concern. Karnataka, a southern state in India, faces a significant burden of mosquito-borne diseases including malaria, dengue, lymphatic filariasis, and Japanese encephalitis, with the prevalence varying across districts. In 2010, Karnataka reported 1,09,118 malaria cases, 28,065 of which were attributed to Plasmodium falciparum. ^13^

The coastal cities of Mangalore and Udupi together account for approximately 72% of the malaria cases reported in Karnataka. ^14^ In particular, Mangalore—a coastal town in the Dakshina Kannada district with frequent heavy rainfall and high humidity—provides an ideal environment for mosquito proliferation. An entomological survey conducted in Mangalore taluk identified 26 mosquito species across six genera, with an annual parasite index of 10–12, indicating that the area was malaria endemic. ^15^ Rapid urbanization in urban Mangalore, including extensive construction, inadequate drainage, and poor road conditions, has further contributed to the persistence of the endemic nature of mosquito-borne diseases in the area. ^16^

Numerous studies have identified environmental, socioeconomic, and behavioral determinants of mosquito proliferation and vector-borne disease transmission. Aquatic habitats, such as pools, streams, and water-filled containers, are critical breeding sites for mosquitoes. While many *Anopheles* species breed in relatively clean, fresh water, this is not universal. Notably, the urban vector *Anopheles stephensi*, which has become invasive in several regions including the Arabian Peninsula and the Horn of Africa, is well adapted for breeding in domestic water containers and polluted urban habitats. ^17^ It is also the predominant urban malaria vector in many parts of India, including coastal Karnataka. ^18^ Furthermore, behavioral adaptations among *Anopheles* species have been documented in response to vector control interventions, with some species shifting toward earlier or greater diurnal biting activity following indoor residual spraying (IRS) and the deployment of long-lasting insecticidal nets (LLINs). ^19^

A study by Wilke et al. (2020) in Miami-Dade Florida identified a few of the common aquatic habits that are responsible for harboring 80% of all immature *Ae. Aegypti* and increasing their proliferation in the presence of those aquatic habitats. ^20^ Similarly, Prashanthi *et al.* (2007) reported that *Anopheles* breeds in pools and streams, where people living in close proximity are at high risk of malaria and its transmission. ^21^ These studies have also revealed that socioeconomic factors exacerbate the vulnerability to malaria, as economically marginalized populations often lack access to anti-mosquito measures, such as mosquito nets or repellents, and may follow age-old traditional practices, such as sleeping outdoors at night amid peak mosquito activity.

The use of preventive measures, such as effective lids over water storage containers and frequent emptying of containers, significantly reduces the incidence of arthropod proliferation, especially in *Ae. aegypti.* ^22^ Studies have shown a linear relationship between growing populations, rising socioeconomic status, and increased mosquito proliferation, particularly in economically marginalized densely populated areas vulnerable to dengue. ^23^ A study by Srividya *et al.* (2018), through logistic regression analyses, indicated that tiled and concrete dwellings increased the likelihood of an area becoming a dengue hotspot by 2.0 and 2.9 times, respectively, due to the conducive breeding environment. ^24^

There is a significant relationship between rapid, unplanned urbanization and the proliferation of mosquitoes. Mangalore has experienced dramatic urbanization in recent years, and these unplanned disorganized cities aggravate mosquito proliferation, especially in *Aedes aegypti* by creating artificial breeding grounds, such as stagnant water pools, and increasing disease transmission. ^25^ Climate change also has a considerable effect on vector proliferation. It reduces larval development time and rapidly increases mosquito populations. This also leads to a reduction in the extrinsic incubation period of pathogens in mosquitoes, thereby increasing their infectiousness. ^25^

Community knowledge and behavior are critical for effective vector control. A study by Garbin et al. (2021) revealed that while 76% of respondents believed that their neighborhood was likely to be infected by a disease spread by mosquitoes, but no action was taken by them, highlighting a gap between awareness and actions. ^26^ Another study by Madeira et al. (2002) demonstrated that didactic interventions among schoolchildren increased knowledge about mosquito breeding sites and vector proliferation, leading to heightened awareness. ^27^

Various determinants of mosquito proliferation have been identified across different studies, and the present study builds on this evidence by exploring the specific environmental, socioeconomic, and behavioral factors driving mosquito proliferation in Mangalore and assessing community measures to mitigate vector-borne disease risk. By examining vector nuisance, disease prevalence, and community engagement, this study aligns with Sustainable Development Goal 3.3—ending epidemics of malaria and other communicable diseases.

## Objectives

1. To identify the key environmental, socioeconomic, and behavioral determinants associated with mosquito presence and density within the study setting.
2. To assess the community-level preventive measures adopted to reduce mosquito breeding and control mosquito-borne diseases.
3. To evaluate community perceptions, attitudes, and practices regarding mosquito-borne diseases.
4. To estimate the self-reported burden of mosquito-borne diseases among the study population.

## Methodology

**Figure 1.**
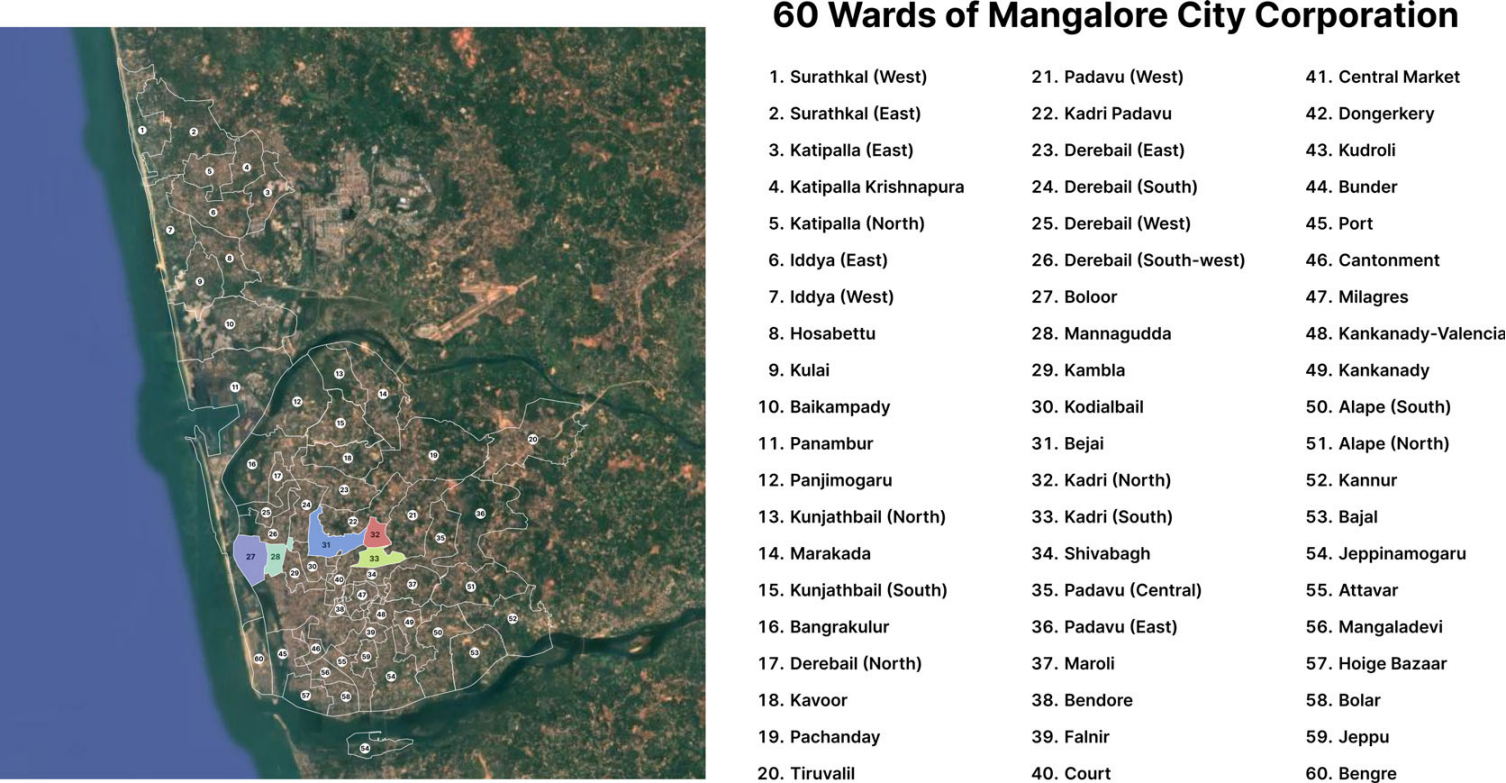
Map showing the Mangalore City Corporation study areas consisting of 60 wards.

This community-based cross-sectional study was conducted in Mangalore, a coastal city on the western coast of Karnataka, a South Indian state. Mangalore with an area of 132.4 km ^2^ is situated between 12°50′30″ N to 13°01′00″ N and 74°48′0″ E to 74°55′00″ E coordinates, is a tropical river basin, and has a humid climate of peninsular India. Mangalore is bounded by the western Ghats to the east, the Arabian Sea to the west, Kerala to the south, and the Udupi district to the north. Mangalore, the district headquarters of Dakshina Kannada, is administered by a city corporation founded in 1865 and consists of 60 wards. ^28^ Wards 27, 28, 31, 32 and 33 were chosen as study areas ( Figure 1). According to the Census of India 2011, the population of the Mangalore City Corporation was 499,487. In the absence of a more recent official census, population projections indicate that it may have grown to approximately 700,000 residents by 2024. ^29^

The study was conducted between September and October 2024. The sample size was calculated based on a previous study conducted in Mangalore, Karnataka, ^30^ which reported that 83% of the people used preventive measures such as mosquito nets to prevent mosquito bites, using this as our anticipated proportion and 10% relative precision, 97.5% quantile of the standard normal distribution, and considering 20% non-response rate as 95 sample size was calculated as follows:

where *p* = 83%, *d* = 10% of 83% = 8.3%, *Z* = 1.96

$$n=\frac{Z_{1-\frac{a}{2}}^{2}P\left(1-P\right)}{d^{2}}$$

$$n=\frac{1.96^{2}0.83\left(1-0.83\right)}{0.083^{2}}$$

$$n=\frac{0.5421}{0.006889}=78$$

To account for 20% of the non-responses, 95 households were selected.

The study protocol was approved by the Institutional Ethics Committee, Kasturba Medical College Mangalore with No: IEC KMC MLR 09/2024/587, followed by permission from the Head of the Institute. The written consent was taken on an ‘Informed consent form’ which was provided to the participants ≥18 years of age for signature, and the participants <18 years of age were excluded from the study. All participants had the right to withdraw at any stage of the study, and all incomplete responses were considered withdrawal and excluded from the analysis. The study participants were approached from a household that was selected from five wards out of the total 60 wards present under the Mangalore City Corporation on the basis of convenience; one reliable informant residing in the household for more than at least 1 year who was aware of the household conditions and consented was included in the study, whereas temporary residents of less than a year, apartment complexes, and households without adult personnel were excluded.

The number of households included in our study was divided equally among each ward; that is, a total of 19 households from each of the selected wards were considered for the study. A random street was selected from each ward and then, standing at the end of each randomly selected street, every 3 ^rd^ house present on the left side of the lane was considered for the study, which was continued in a clockwise order; in case of failure to meet the inclusion criteria, absence, or denial to take part in the study, the next house was considered. When the head of the household was absent during the time of data collection, information was collected from the oldest adult residing in the household.

A pretested, semi-structured, and internally validated questionnaire was developed for data collection. The questionnaire was constructed following an extensive literature review using electronic databases such as PubMed, Scopus, and Google Scholar, covering studies published between 2000 and 2024 that examined mosquito ecology, vector proliferation and nuisance, community perceptions, preventive practices and self-reported mosquito-borne illnesses in urban settings. Relevant national guidelines from the National Vector Borne Disease Control Programme (NVBDCP) were also reviewed to ensure contextual alignment. ^31, 32^

Content validation was conducted by a panel of three public health specialists and two medico social workers—from the Department of Community Medicine, Kasturba Medical College, Mangalore. Each expert independently assessed the questionnaire items for clarity, relevance, simplicity, and ambiguity using a four-point Likert scale (1 = not relevant, 4 = highly relevant). The item-level content validity index (I-CVI) was calculated as the proportion of experts rating each item as either 3 or 4. Items with an I-CVI less than 0.78 were revised or removed on the basis of the panel’s feedback. The scale-level content validity index (S-CVI/Ave), computed as the average of all the I-CVIs, score ≥ 0.8 was considered satisfactory, indicating good overall content validity.

The data were recorded after informed consent was obtained from the head of households. The questionnaire was self-administered; however, for illiterate participants, it was administered by the investigators. The questionnaire was designed to assess community perceptions of mosquito nuisance and other determinants. Accordingly, participant response categories such as mild and moderate to severe were used to capture the perceived intensity of mosquito nuisance compared with the previous year and may not fully capture reduced nuisance levels. The data collected were entered into MS Excel and analyzed using Jamovi version 2.6.26. Descriptive statistics are presented as frequencies and proportions. Confidence intervals (95%) for proportions were computed using the exact binomial method. The association between two categorical variables was assessed using the chi-square test. The strength of the association between the independent and dependent variables were measured using odds ratios (OR) with corresponding 95% confidence intervals (CI) and significant *p* value of <0.05. Variables with p value <0.2 in the univariate logistic regression were included in the multivariate model. To adjust for familywise error rate (FWER), Holms correction was applied to the *p* values.

## Results

The study included 95 households; the majority of the participants were above the age of 45 years (72.6%), and most were females (70.5%). The majority of participants (94.8%) were educated and 61.1% reported having access to a healthcare facility within 2 km of their residence ( Table 1).

**Table 1.** Socio-demographic characteristics of study participants (N = 95).

Socio-demographic details	n (%)	95% CI
**Age (in years)**		
18-30	12 (12.6)	6.70% - 21.03%
31-45	14 (14.7)	8.30% - 23.50%
46-60	34 (35.8)	26.21% - 46.30%
>60	35 (36.8)	27.20% - 47.40%
**Gender**		
Male	28 (29.5)	20.60% - 39.71%
Female	67 (70.5)	60.30% - 79.44%
**Education**		
Illiterate	5 (5.2)	1.73% - 11.90%
Primary School	11 (11.6)	5.92% - 19.80%
High School + PUC	46 (48.4)	38.04% - 58.90%
Degree	33 (34.7)	25.30% - 45.20%
**Proximity of Nearby Health Care facility (in km)**		
0-2	58 (61.1)	50.50% - 70.90%
>2-4	25 (26.3)	17.81% - 36.40%
>4	12 (12.6)	6.70% - 21.03%

Among the study participants, 42.1% reported an increase in mosquito breeding sites past year. Most participants perceived a moderate to severe mosquito presence both indoors (67.4%) and outdoors (87.4%). The evening hours (4–8 pm) were identified as the peak biting time by 71.6% of the respondents. Mosquito nuisance was most prominent during the rainy season (69.5%), and 30.5% reported disturbed sleep or discomfort due to mosquito bites at night ( Table 2).

**Table 2.** Perceived mosquito proliferation and nuisance (N = 95).

Perception	n (%)	95% CI
**Increase in mosquito breeding sites in the last 1 year (yes)**	40 (42.1)	32.04% - 52.70%
**How would you rate the presence of mosquitoes inside the house?**		
Mild	31 (32.6)	23.40% - 43.02%
Moderate to Severe	64 (67.4)	57.0% - 76.64%
**How would you rate the presence of mosquitoes outside the house?**		
Mild	12 (12.6)	6.70% - 21.03%
Moderate to Severe	83 (87.4)	79.00% - 93.0%
**Time of the day when mosquitoes mostly bite**		
Morning (6-10 am)	8 (8.4)	3.71% - 15.92%
Evening (4-8 pm)	68 (71.6)	61.40% - 80.40%
Night (After 8 pm)	19 (20.0)	12.50% - 29.50%
**Mosquito bites causing disturbed sleep or discomfort at night**	29 (30.5)	21.50% - 40.82%
**Weather conditions in which mosquitoes are mostly prevalent**		
Rainy season	66 (69.5)	59.20% - 78.51%
Hot weather	16 (16.8)	9.94% - 25.90%
No noticeable difference	13 (13.7)	7.50% - 22.30%
**Overall mosquito nuisance in locality compared to past one year**		
Mild	38 (40.0)	30.10% - 50.60%
Moderate to Severe	57 (60.0)	49.44% - 69.92%

Water stagnation 74.7% and dense vegetation 54.7% were the most commonly reported determinants of mosquito breeding. Other contributing factors included nearby water bodies (20%) and garbage dumping (13.8%) ( Table 3).

**Table 3.** Determinants of mosquito breeding (N = 95).

Determinants of mosquito breeding *	n (%)	95% CI
**Water stagnation (n = 71)**	71 (74.7)	64.80% - 83.10%
Puddles	53 (74.6)	62.92% - 84.23%
Flowerpots	60 (84.5)	74.0% - 92.0%
Construction sites	23 (32.4)	21.80% - 44.10%
**Garbage Dumping**	13 (13.7)	7.50% - 22.30%
**Presence of water body**	19 (20.0)	12.50% - 29.50%
**Presence of dense vegetation**	52 (54.7)	44.20% - 65.0%
**Water storage in uncovered containers**	12 (12.6)	6.70% - 21.03%
**Presence of water leaks or overflow from pipes and tanks**	8 (8.4)	3.71% - 15.92%

* Multiple responses.

Most households reported using chemical measures such as sprays, vaporizers, or coils (91.6%), closing doors and windows (81.1%), and using mosquito nets or screens (55.8%). Approximately half (51.6%) cleaned their surroundings daily, whereas 38.9% checked for water stagnation weekly. However, 58.9% stated that the municipality rarely conducts cleaning activities, and 50.5% reported no anti-mosquito fogging by local authorities ( Table 4).

**Table 4.** Behavioural and preventive measures by participants for mosquito control (N = 95).

Preventive measures		n (%)	95% CI
**Type of measure** *			
Chemical		87 (91.6)	84.10% - 96.30%
Mosquito nets, window screens or meshes		53 (55.8)	45.23% - 66.0%
Closing doors and windows		77 (81.1)	71.72% - 88.40%
Other personal measures		15 (15.8)	9.12% - 24.70%
**Mosquito repellent/coils**			
Yes, daily		24 (25.3)	16.91% - 35.22%
Yes, occasionally		23 (24.2)	16.01% - 34.10%
Never		48 (50.5)	40.10% - 61.0%
**Insect repellent before sleep**			
Yes		12 (12.6)	6.70% - 21.03%
No		83 (87.4)	79.0% - 93.30%
**Cleaning of surroundings**	**By household members**		
	Daily	49 (51.6)	41.10% - 62.0%
	Weekly	31 (32.6)	23.40% - 43.02%
	Occasionally	15 (15.8)	9.12% - 24.70%
	**By municipality**		
	Yes	39 (41.0)	31.10% - 51.62%
	No	56 (58.9)	48.40% - 68.94%
**Water stagnation (Checking and eliminating stagnation)**			
Daily		17 (17.8)	10.80% - 27.10%
Weekly		37 (38.9)	29.11% - 49.60%
Occasionally		30 (31.6)	22.42% - 41.92%
Never		11 (11.6)	5.92% - 19.80%
**Check holes in window screens/mosquito nets**			
Regularly (monthly/more)		24 (25.2)	16.91% - 35.22%
Occasionally		17 (17.9)	10.80% - 27.10%
Rarely		17 (17.9)	10.80% - 27.10%
Never		37 (39.0)	29.11% - 49.50%
**Anti-mosquito fogging by local authorities**			
Frequently ^#^		6 (6.3)	2.40% - 13.24%
Occasionally		41 (43.1)	33.03% - 53.72%
Never		48 (50.5)	40.10% - 61.0%

* Multiple responses.

^#^ Frequently: weekly or monthly.

Awareness of mosquito-borne diseases was high (92.6%), with malaria (96.6%) and dengue (93.2%) being the most commonly recognized diseases. Most participants (96.8%) believed that education and awareness campaigns are crucial for disease prevention. A majority (83.1%) identified both clean and dirty water as potential breeding sources, and 81.2% noted that mosquito proliferation peaks during the rainy season ( Table 5).

**Table 5.** Awareness and perception of mosquito borne diseases and mosquito control measures (N = 95).

Awareness and perception	n (%)	95% CI
**Awareness about mosquito borne disease** *	88 (92.6)	85.41% - 97.0%
Malaria	85 (96.6)	90.40% - 99.3%
Dengue	82 (93.2)	85.8% - 97.5%
Chikungunya	29 (33)	33.0% - 43.8%
Zika	8 (9.1)	4.01% - 17.13%
Filariasis	9 (10.2)	4.80% - 18.53%
**Perception of Preventive measures to be taken to avoid Mosquito-borne diseases** *		
Regular cleaning of surroundings	76 (80.0)	70.54% - 87.51%
Eliminating stagnant water	71 (74.7)	64.80% - 83.10%
Usage of insecticide sprays	50 (52.6)	42.12% - 63.0%
Usage of mosquito nets	46 (48.4)	38.04% - 68.90%
Education and spreading awareness	92 (96.8)	91.10% - 99.34%
**Perception of community on determinants of mosquito proliferation**		
**a) Water stagnation**		
Clean water	16 (16.8)	9.94% - 25.90%
Dirty water	44 (46.3)	36.02% - 56.84%
Both clean and dirty water	35 (36.8)	27.20% - 47.40%
**b) Seasonal variation**	85 (89.5)	81.50% - 94.84%
Rainy season	69 (81.2)	71.24% - 88.84%
Summer season (Dry Hot weather)	16 (18.8)	11.20% - 28.80%

* Multiple responses.

In the past year, 29.4% of the participants reported suffering from a mosquito-borne disease, primarily dengue (71.4%) and malaria (28.5%). Common symptoms included fever (96.4%), headache (78.5%), joint pain (60.7%), and muscle pain (46.4%). Most sought treatment at private clinics (82.2%). Post recovery complications such as generalized weakness were reported by 92.3% of the patients ( Table 6).

**Table 6.** Self-reported cases and outcomes of mosquito borne diseases (N=95).

Self-Reported cases	N (%)	95% CI
**Suffered from any mosquito-borne disease in the past 1 Year**		
Yes	28 (29.4)	20.60% - 39.71%
**Mosquito borne Disease (N = 28)**		
Malaria	8 (28.5)	13.22% - 48.70%
Dengue	20 (71.4)	51.33% - 86.80%
**Symptoms** * **(N = 28)**		
Fever	27 (96.4)	81.70% - 99.91%
Headache	22 (78.5)	59.10% - 91.70%
Joint Pain	17 (60.7)	40.60% - 78.50%
Rash	2 (7.1)	1.8% - 23.50%
Muscle Pain	13 (46.4)	27.51% - 66.13%
Others	17 (60.7)	40.60% - 78.50%
**Place of Treatment (N = 28)**		
Public Health Centre	5 (17.8)	6.1% - 36.9%
Private Clinic	23 (82.2)	63.11% - 93.94%
**Complications after recovery** * **(N = 13)**		
Weaknesses	12 (92.3)	64.0% - 99.8%
Cold	1 (7.6)	0.20% - 36.03%
Leg Pain	1 (7.6)	0.20% - 36.03%
Headache	1 (7.6)	0.20% - 36.03%
Eye Pain	1 (7.6)	0.20% - 36.03%

* Multiple choice question.

Variables with p value <0.2 in the univariate logistic regression were included in the multivariate model. After applying Holm’s correction for the familywise error rate (FWER), the logistic regression model showed no significant associations between mosquito density and factors such as water stagnation, construction sites, nearby water bodies, dense vegetation, garbage dumping, type of water storage, or water leaks ( Table 7).

**Table 7.** Associations of environmental factors with mosquito density: univariate and multivariate logistic regression analysis (N=95).

Variable	Increased mosquito density		Unadjusted OR (95% CI)	p value *	Adjusted OR (95% CI)	p value *
	Yes (%) n = 49	No (%) n = 46				
Presence of water stagnation Yes No	34 (69.3) 15 (30.6)	22 (47.8) 24 (52.1)	2.5 (1.1, 5.7) - -	0.198 ^#^ - -	2.1 (0.9, 5) - -	0.097 - -
Presence of construction sites Yes No	17 (34.6) 32 (65.3)	6 (13.0) 40 (86.9)	3.5 (1.2, 10.0) - -	0.098 ^#^ - -	3 (1, 8.8) - -	0.084 - -
Presence of any water body (pond, lake, etc.) Yes No	8 (16.3) 41 (83.6)	7 (15.2) 39 (84.7)	1.1 (0.4, 3.3) - -	0.882 - -	- - -	- - -
Presence of Dense Vegetation Yes No	31 (63.2) 18 (36.7)	21 (45.6) 25 (54.3)	2.1 (0.9, 4.6) - -	0.425 - -	- - -	- - -
Presence of Garbage dumping sites Yes No	8 (16.3) 41 (83.6)	5 (10.8) 41 (89.1)	1.6 (0.5, 5.3) - -	0.878 - -	- - -	- - -
Type of Water storage Covered containers Uncovered containers	41 (83.6) 8 (16.3)	42 (91.3) 4 (8.6)	2.1 (0.6, 7.3) - -	0.789 - -	- - -	- - -
Presence of water leaks or overflow from tanks or taps Yes No	6 (12.2) 43 (87.7)	2 (4.3) 44 (95.6)	3.1 (0.6, 16.1) - -	0.664 - -	- - -	- - -

OR = Odds Ratio; CI = Confidence Interval.

* Holms corrected p value.

^#^ (p value ≤ 0.2; χ ^2^ test).

The use of mosquito repellents or coils was paradoxically associated with a higher incidence of mosquito-borne diseases (OR = 3.7; 95% CI: 1.4–9.6; p = 0.024), suggesting that repellent use may be more common in areas with higher mosquito density than effective preventive measure ( Table 8).

**Table 8.** Association between the mosquito borne disease prevalence and preventive measures taken inside the house (N = 95).

Variable	Presence of mosquito-borne disease		OR (95 CI%)	p Value *
	Yes (%) n = 28	No (%) n = 67		
Repair or check holes in window screens or mosquito nets Yes No	9 (32.1) 19 (67.8)	32 (47.7) 35 (52.2)	0.5 (0.2, 1.2) - -	0.483 - -
Use of mosquito repellents or coils Yes No	20 (71.4) 8 (28.5)	27 (40.3) 48 (59.7)	3.7 (1.4, 9.6) -	0.024 ^#^ - -
Use of electric mosquito bats or insecticide sprays Often Rarely	11 (39.2) 17 (60.7)	22 (32.8) 45 (67.1)	1.3 (0.5, 3.3) -	>0.999 - -
Usage of Mosquito nets at night Yes No	14 (50.0) 14 (50.0)	37 (55.2) 30 (44.7)	0.8 (0.3, 1.9) -	>0.999 - -

OR = Odds Ratio; CI = Confidence Interval.

* Holms corrected p value.

^#^ Statistically significant (p value ≤ 0.05; χ ^2^ test).

## Discussion

The present study, which was conducted within the Mangalore City Corporation, provides valuable insights into the determinants of mosquito population density and community responses in the urban setting of coastal Karnataka. These findings affirm that urban mosquito breeding and disease transmission are influenced by a complex interplay of environmental, behavioral, and infrastructural factors.

In the present study, a majority (95%) had some level of education and were above 45 years of age, with a predominance of female respondents, and 61% reported nearby healthcare facilities within 2 km, indicating relatively good access to healthcare in the study area. These findings match those of prior studies highlighting that educational level and proximity to health services may influence awareness and preventive behaviors related to vector-borne diseases, although not necessarily with consistent environmental control practices. ^26, 27^

More than two-fifths of the respondents perceived an increase in mosquito breeding sites within the last year and 60% of participants reported experiencing moderate to severe mosquito nuisance in their locality over the past year, which was consistent with urbanization-related ecological changes in the study setting. This aligns with the national trends of urban vector expansion reported in the National Vector Borne Disease Control Programme (NVBDCP) surveillance data. ^32^ Notably, 71.5% of the participants indicated that evenings were the peak time for mosquito activity, and 30.5% reported that mosquito bites disrupted their sleep, which is consistent with *Aedes aegypti’s* day-biting habits, whereas *Anopheline* mosquitoes are known for nocturnal activity, which suggests the presence of mixed mosquito species in the study area. ^21^

Additionally, 67.4% of the participants described a moderate to severe presence of mosquitoes indoors, whereas 87.3% reported similar conditions outdoors. This finding is comparable to data from Kampango et al., who reported an average of 85.93% An. gambiae s.l. bites per night, with 66% occurring indoors and 34% outdoors, peaking between 22:00 and 03:00 in a rural community in Chókwè District, southern Mozambique. ^33^

In terms of environmental factors, 69.4% of the participants reported high mosquito activity during the rainy season, which corresponds to the behavioral patterns of *Aedes aegypti* and *Culex quinquefasciatus*, both of which are well adapted to peridomestic environments. ^1^ This aligns with a cross-sectional study by Mahgoub et al. in Barakat and El-Kareiba, Sudan, which reported a high number of positive habitats during the rainy season, whereas the lowest numbers were reported during the hot season followed by the dry season, corroborating findings that climate and seasonal variation significantly influence mosquito density and disease transmission. ^25, 34^

The primary breeding sites identified in our study included water stagnation (74.7%), dense vegetation (54.7%), and nearby water bodies (20%), which is consistent with previous studies demonstrating the importance of stagnant water and vegetation in vector proliferation. ^20^^–^^22^ Urban infrastructure projects, especially when poorly managed, contribute to temporary water stagnation, while dense vegetation offers resting sites and microclimatic conditions favourable to adult mosquitoes.

After applying Holm’s correction for the familywise error rate (FWER), the logistic regression model revealed no significant associations between mosquito density and environmental factors such as water stagnation (OR 2.5, 95% CI: 1.1, 5.7, p value – 0.198), construction sites (OR 3.5, 95% CI: 1.2, 10.0, p value – 0.098), nearby water bodies (OR 1.1, 95% CI: 0.4, 3.3, p value – 0.882), dense vegetation (OR 2.1, 95% CI: 0.9, 4.6, p value – 0.425), garbage dumping (OR 1.6, 95% CI: 0.5, 5.3, p value – 0.878), type of water storage (OR 2.1, 95% CI: 0.6, 7.3, p value – 0.789), or water leaks (OR 3.1, 95% CI: 0.6, 16.1, p value – 0.664). Water stagnation near residential compounds may often result from inadequate municipal drainage systems—a structural determinant beyond individual household control. ^24, 25^

Preventive measures among participants were notable, as 91.5% used chemical repellents (insect sprays, vaporizers, and smoke), 81% kept doors and windows closed, 55.7% utilized physical barriers such as mosquito nets or screens, and 15.7% employed other personal measures. These findings are consistent with household-level studies from Mumbai and Sri Lanka, which reported that city respondents, on the other hand, were more likely to use liquid repellents and mosquito sprays, perhaps owing to their ease of use and their immediate, visible effects and commercial availability. ^35, 36^

In terms of community cleaning practices, 51.5% of individuals reported cleaning their surroundings daily; additionally, 38.9% checked for water stagnation weekly; in contrast, 84.6% never reported water stagnation to authorities. Unfortunately, 58.9% indicated that municipal cleaning was infrequent in the locality, reflecting gaps in sustained vector management. Notably, 25.2% of the participants regularly checked window screens or mosquito nets for damage, and 50.5% stated that there had been no anti-fogging efforts by local authorities, underscoring the need for better municipal participation and routine surveillance-based larval control, as emphasized in the National Framework for Malaria Elimination 2016–2030. ^31^ This is in contrast with Mahalakshmi et al.’s findings in northern Gujarat, where 88% cleaned their homes daily, 57.3% cleaned their surroundings weekly, and 82% actively avoided water stagnation. ^37^

High awareness of mosquito-borne diseases (92.6%), particularly malaria and dengue, is comparable to that reported in other urban studies. However, awareness alone does not guarantee effective preventive action, a well-documented paradox in vector control studies. For example, a community-based survey from Puducherry revealed that although 85.5% of the total respondents had heard of dengue fever and that most of them (82.7%) were aware that it is transmitted through mosquito bites, only 25.1% of participants were aware that the dengue mosquito breeds in clean water-holding containers. ^38^

Despite these measures, approximately 30% of the participants reported suffering from a mosquito-borne disease in the past year, with dengue accounting for 71.4% of the cases and malaria accounting for (28.6%). Dengue virus consists of four antigenically distinct serotypes (DENV-1 to DENV-4), and the cocirculation of multiple serotypes such as DENV-1, DENV-2, and DENV-3 contributes to complex transmission patterns. Primary infection confers only short-term cross-protection, and secondary infection with a heterologous serotype is well known to carry a markedly increased risk of severe disease due to mechanisms such as antibody-dependent enhancement. Consequently, such clinically overt secondary infections are more likely to be detected and reported, which may explain the higher proportion of dengue cases observed in the study setting. ^39^

Common symptoms included fever (96.4%), headache (78.5%), joint pain (60.7%), and muscle pain (46.4%), which aligns with the WHO’s case definition for dengue and aligns with findings from a study conducted by Kumar et al. in a tertiary hospital in the Udupi district, which indicated that 83.9% of cases were due to dengue fever, presenting symptoms such as fever (99.1%), myalgia (64.6%), vomiting (47.6%), headache (47.6%), abdominal pain (37.5%), and breathlessness (17.8%). ^9, 40^

In this study, 92.3% of the participants reported experiencing complications post recovery, suggesting prolonged morbidity, an often underrecognized component of dengue disease burden. This contrasts with a study in Vietnam by Tam et al., who reported that 12.5% of participants experienced alopecia, 11.1% had blurred vision, 9.5% faced concentration difficulties, and 8.5% suffered from fatigue following dengue infection. ^41^

The present study revealed that 82.1% of the population perceives water stagnation as a significant factor in mosquito proliferation, indicating that it is a major factor in the prevalence of mosquito-borne diseases. Construction sites, water bodies, dense vegetation, and water storage in open containers were identified as significant determinants. Additionally, 17.8% of individuals perceive water leakage from tanks and taps as key determinants, indicating that water leakage from household stores is a significant factor in the prevalence of mosquito-borne diseases. Overall, these factors contribute to the prevalence of mosquito-borne diseases.

Interestingly, the use of mosquito repellents or coils was significantly associated with a greater incidence of mosquito-borne diseases (OR 3.7, 95% CI: 1.4, 9.6, p = 0.024) suggesting a reactive response: — households in high-risk areas or with prior illness episodes are more likely to adopt repellents. This phenomenon has been described in behavioral epidemiology as the “reverse causation effect”. ^42^ This finding also suggests possible improper usage or overreliance on repellents without addressing environmental breeding sites. Similar gaps between knowledge and action have been reported in prior studies. ^26^

## Limitations

As a cross-sectional study, temporal causality between determinants and mosquito density could not be established. Entomological indices (e.g., the Breteau or House index) were not measured, limiting direct quantification of vector density. Self-reported disease history may be subject to recall bias, although the one-year recall period likely limits its magnitude. This study did not assess local insecticide resistance patterns, which is relevant given the presence of *Anopheles stephensi* in the region. Nonetheless, this study provides strong evidence linking environmental and behavioral factors to perceived mosquito proliferation, guiding targeted urban health interventions.

## Conclusion

In conclusion, this study provides an integrated approach by identifying key environmental determinants of mosquito presence while simultaneously evaluating community preventive measures, perceptions and self-reported mosquito-borne disease burden, offering context specific insights from Mangalore that complement previous studies in other settings. While knowledge and attitudes in the community were generally adequate, their association with actual preventive practices was modest, indicating a persistent gap between awareness and action. However, proper intervention by local authority is necessary to combat the main environmental factors responsible for mosquito breeding. This highlights the gaps found in our study, where despite widespread awareness of mosquito-borne diseases, respondents acknowledged limited knowledge of effective preventive measures and exhibited suboptimal preventive practices. Hence, in addition to awareness, there is a dire need to provide the right personnel and services to combat mosquito-borne diseases.

## Recommendation

### Awareness and campaigns

Nearly one hundred percent of the participants believed that spreading awareness of mosquito-borne diseases and mosquito control measures would help reduce the incidence of mosquito borne diseases in the community. This can be accomplished through various means, such as public service announcements, social media campaigns, and community outreach programs.

### Elimination of water stagnation

Local authorities and communities must proactively repair damaged roads, cover open drains, strengthen vector control at construction sites, ensure proper waste disposal to eliminate standing water, prevent mosquito breeding, and reduce the risk of vector-borne diseases.

### Clearing up of dense vegetation

Dense vegetation was identified as a significant determinant of mosquito presence in household neighborhoods. Local authorities should ensure regular clearing of vegetation and maintenance of cleanliness in these areas.

### Further research

Further research to identify the factors leading to an increase in the mosquito population and community and local government measures can help identify additional intervention strategies. This can involve qualitative research methods, such as in-depth interviews, field research, or focus groups, to gain a deeper understanding of the underlying issue.

## Ethical approval

The study protocol was approved by the Institutional Ethics Committee, Kasturba Medical College Mangalore with No: IEC KMC MLR 09/2024/587, followed by permission from the Head of the Institute. The written consent was taken on an ‘Informed consent form’ which was provided to the participants ≥18 years of age for signature, and the participants <18 years of age were excluded from the study. All participants had the right to withdraw at any stage of the study, and all incomplete responses were considered withdrawn and excluded from the analysis.

## Data Availability

The data set used and/or analyzed during the current study are available from the online repository (figshare) DOI- https://doi.org/10.6084/m9.figshare.29144975 ^43^ (Tables 1–8). The data include participant information sheet, Informed consent form and questionnaire are available from the online repository (figshare) DOI- https://doi.org/10.6084/m9.figshare.29144990. ^44^ Data are available under the terms of the Creative Commons Zero “No rights reserved” data waiver (CC0 Public domain dedication).

## References

[1] WHO: *Global vector control response 2017–2030.* Geneva: World Health Organization;2017.

[2] World Health Organization : *Vector-borne diseases: key facts [Internet].* Geneva: World Health Organization;2024[cited 2025 Nov 10]. Reference Source

[3] Venkatesan P : The 2023 WHO World malaria report. *Lancet Microbe.* 2024 Mar 1;5(3):e214. 10.1016/S2666-5247(24)00016-8 38309283

[4] World Health Organization : *World Malaria Report 2023.* Geneva: WHO;2023.

[5] WHO Regional Office for South-East Asia : *Malaria Situation in the South-East Asia Region 2023.* New Delhi: WHO-SEARO;2023.

[6] National Center for Vector Borne Disease Control: *Operational guidelines for hospitals to control Aedes breeding.* New Delhi: National Center for Vector Borne Diseases Control;2022. Reference Source

[7] Bhatt S Gething PW Brady OJ : The global distribution and burden of dengue. *Nature.* 2013;496(7446):504–507. 10.1038/nature12060 23563266 PMC3651993

[8] Brady OJ Gething PW Bhatt S : Refining the global spatial limits of dengue virus transmission by evidence-based consensus. *PLoS Negl. Trop. Dis.* 2012;6(8):e1760. 10.1371/journal.pntd.0001760 22880140 PMC3413714

[9] World Health Organization : *Dengue and severe dengue: Key facts.* Geneva: WHO;2024. Reference Source

[10] Global dengue surveillance : *World Health Organization.* [January 02, 2025]. Reference Source

[11] Paulson W Kodali NK Balasubramani K : Social and housing indicators of dengue and chikungunya in Indian adults aged 45 and above: Analysis of a nationally representative survey (2017-18). *Arch. Public Health.* 2022;80:125. 10.1186/s13690-022-00868-5 35443704 PMC9022351

[12] Chavasse D : Know your enemy: Some facts about the natural history of Malawi’s Anopheles mosquitoes and implications for malaria control. *Malawi Med. J.* 2002 Apr 1;14(1):7–8. 27528915 PMC3345420

[13] Kumar KR Gururaj G : Community Perception Regarding Mosquito-borne Diseases in Karnataka State, India.

[14] Prasad KI Govindarajan R Sreepada KS : Seasonal Diversity of mosquito species in Dakshina Kannada district, Karnataka, India. *Journal of Vector Borne Diseases.* 2021 Apr 1;58(2):119–125. 10.4103/0972-9062.321758 35074945

[15] Dayanand KK Punnath K Chandrashekar V : Malaria prevalence in Mangaluru city area in the southwestern coastal region of India. *Malar. J.* 2017 Dec;16:1. 10.1186/s12936-017-2141-0 29258505 PMC5735873

[16] Ghosh SK Manju R : Malaria elimination in India—The way forward. *J. Vector Borne Dis.* 2019;56(1):32–40. 10.4103/0972-9062.257771 31070163

[17] WHO : *Anopheles stephensi: global threat in urban malaria transmission.* Geneva: WHO;2023.

[18] Ghosh SK Tiwari S Raghavendra K : Observations on sporozoite detection in naturally infected sibling species of the Anopheles culicifacies complex and variant of Anopheles stephensi in India. *J. Biosci.* 2008 Sep;33(3):333–336. 10.1007/s12038-008-0052-5 19005232

[19] Russell TL Lwetoijera DW Maliti D : Impact of promoting longer-lasting insecticide treatment of bed nets upon malaria transmission in a rural Tanzanian setting with pre-existing high coverage of untreated nets. *Malar. J.* 2010 Jun 28;9(1):187. 10.1186/1475-2875-9-187 20579399 PMC2902500

[20] Wilke AB Vasquez C Carvajal A : Proliferation of Aedes aegypti in urban environments mediated by the availability of key aquatic habitats. *Sci. Rep.* 2020 Jul 31;10(1):12925. 32737356 10.1038/s41598-020-69759-5 PMC7395141

[21] Prashanthi D Ranganathan CR Balasubramanian S : Socio-demographic determinants of Malaria in highly infected rural areas: regional influential assessment using GIS. GIS for Health and the Environment: Development in the Asia-Pacific Region With 110 Figures. 2007;195–205.

[22] Yadav K Dhiman S Rabha B : Socio-economic determinants for malaria transmission risk in an endemic primary health centre in Assam, India. *Infect. Dis. Poverty.* 2014 Dec;3:1–8. 10.1186/2049-9957-3-19 24991410 PMC4078389

[23] Telle O Vaguet A Yadav NK : The spread of dengue in an endemic urban milieu–the case of Delhi, India. *PloS one.* 2016 Jan 25;11(1):e0146539. 10.1371/journal.pone.0146539 26808518 PMC4726601

[24] Srividya A Subramanian S Sadanandane C : Determinants of transmission hotspots and filarial infection in households after eight rounds of mass drug administration in India. *Trop. Med. Int. Health.* 2018 Nov;23(11):1251–1258. 10.1111/tmi.13143 30152049

[25] Almeida LS Cota AL Rodrigues DF : Sanitation, Arboviruses, and Environmental Determinants of Disease: impacts on urban health. *Ciencia & saude coletiva.* 2020 Sep 28;25:3857–3868. 10.1590/1413-812320202510.30712018 32997018

[26] Garbin CA Teruel GP Saliba TA : Knowledge and attitude of women with high-risk pregnancy about the Zika virus transmission. *Ciênc. Saúde Colet.* 2021 Jan 25;26:233–240. 10.1590/1413-81232020261.25752018 33533844

[27] Madeira NG Macharelli CA Pedras JF : Education in primary school as a strategy to control dengue: An evaluation in São Paulo State, Brazil. *Rev. Soc. Bras. Med. Trop.* 2002;35:221–226. 10.1590/S0037-86822002000300004 12045814

[28] Kushalan S Kashyap A Morajkar S : Geospatial Distribution of Fluoride and Iron in Natural Water Sources in Mangalore City. *Journal of Health and Allied Sciences NU.* 2023 Oct;13(04):525–534. 10.1055/s-0042-1760322

[29] Mangalore Municipal Corporation City population census 2011-2025 [Internet] . Census2011.co.in. [cited2025 Nov 14]. Reference Source

[30] Maskeri R Jain A Ullal S : Knowledge, Attitude and Practices (KAP) Regarding Malaria and its Prevention among Patients with Suspected Malaria in Mangaluru. *Indian J. Public Health Res. Dev.* 2018 Sep 1;9(9):271–276. 10.5958/0976-5506.2018.01008.2

[31] NVBDCP : National Framework for Malaria Elimination in India 2016–2030. *Directorate General of Health Services, Ministry of Health and Family Welfare.* 2016.

[32] NVBDCP : *Guidelines for Integrated Vector Management in India.* DGHS;2020.

[33] Kampango A Pinto J Abílio AP : Characterisation of human exposure to nocturnal biting by malaria and arbovirus vectors in a rural community in Chókwè district, southern Mozambique. *Wellcome Open Research.* 2023;8:193. 10.12688/wellcomeopenres.19278.1 37484481 PMC10357080

[34] Mahgoub MM Kweka EJ Himeidan YE : Characterisation of larval habitats, species composition and factors associated with the seasonal abundance of mosquito fauna in Gezira, Sudan. *Infect. Dis. Poverty.* 2017 Dec;6:1. 10.1186/s40249-017-0242-1 28173839 PMC5297020

[35] Dhawan G Joseph N Pekow PS : Malaria-related knowledge and prevention practices in four neighbourhoods in and around Mumbai, India: a cross-sectional study. *Malar. J.* 2014 Aug 7;13(1):303. 10.1186/1475-2875-13-303 25102949 PMC4248467

[36] Sānu MN Fernando SD De Silva BN : Use of Household Insecticides against Mosquitoes in Dengue-Endemic Areas in Sri Lanka. *Am. J. Trop. Med. Hyg.* 2024 Jan 23;110(3):549–556. 10.4269/ajtmh.22-0639 38266293 PMC10919195

[37] Mahalakshmi B Sivasubramanian N Vaghela P : Awareness on mosquito borne diseases among urban & rural population in Northern Gujarat. *Bioinformation.* 2022;18(7):640–644. 10.6026/97320630018640 37313054 PMC10259226

[38] Jeelani S Sabesan S Subramanian S : Community knowledge, awareness and preventive practices regarding dengue fever in Puducherry – South India. *Public Health.* 2015 Jun;129(6):790–796. 10.1016/j.puhe.2015.02.026 25863688

[39] Rothman AL : Immunity to dengue virus: a tale of original antigenic sin and tropical cytokine storms. *Nat. Rev. Immunol.* 2011;11(8):532–543. 10.1038/nri3014 21760609

[40] Kumar A Rao CR Pandit V : Clinical manifestations and trend of dengue cases admitted in a tertiary care hospital, Udupi district, Karnataka. *Indian J. Community Med.* 2010 Jul 1;35(3):386–390. 10.4103/0970-0218.69253 21031102 PMC2963875

[41] Tam DT Clapham H Giger E : Burden of postinfectious symptoms after acute dengue, Vietnam. *Emerg. Infect. Dis.* 2023 Jan;29(1):160–163. 10.3201/eid2901.220838 36573590 PMC9796196

[42] Rosenstock IM : The health belief model and preventive health behavior. *Health Educ. Monogr.* 1974;2(4):354–386. 10.1177/109019817400200405 299611

[43] Surendran J Dr : VBD F1000Research data sheet.Dataset. *figshare.* 2025. 10.6084/m9.figshare.29144975

[44] Surendran J Dr : EXTENDED DATA VBD F1000RESEARCH.Dataset. *figshare.* 2025. 10.6084/m9.figshare.29144990

