# Supplementary material for: Determinants of urban mosquito population density and community responses: A cross-sectional study
Source: F1000Res. 2025 Dec 18;14:661. Originally published 2025 Jul 7. [Version 4] doi: 10.12688/f1000research.164704.4 (PMC12701949; doi:10.12688/f1000research.164704.4)
Supplement: Peer review history

# Reviewer report: Reviewer response for version 4

**DOI:** 10.5256/f1000research.193548.r442634

**Published:** 2026-01-02

**Authors:** Asma Rahim

**Affiliations:** 1 Government Medical College, Kozhikode, India

The authors have addressed the points for revision raised by me satisfactorily. This article is fit for indexing.

Is the work clearly and accurately presented and does it cite the current literature?

Partly

If applicable, is the statistical analysis and its interpretation appropriate?

Partly

Are all the source data underlying the results available to ensure full reproducibility?

Yes

Is the study design appropriate and is the work technically sound?

Partly

Are the conclusions drawn adequately supported by the results?

Partly

Are sufficient details of methods and analysis provided to allow replication by others?

Partly

Reviewer Expertise:

Public health , Community Rheumatology, Health professions education

I confirm that I have read this submission and believe that I have an appropriate level of expertise to confirm that it is of an acceptable scientific standard.

# Reviewer report: Reviewer response for version 4

**DOI:** 10.5256/f1000research.193548.r443038

**Published:** 2026-01-02

**Authors:** Oluwaseun Adegbola Adesoye

**Affiliations:** 1 University of Abuja, Abuja, Nigeria

The work is clearly stated and well presented. However, some references cited are too old. For instance, reference 22 and 27 is too old since the work examines recent happening that aids mosquito proliferation. 

 Also, some major statistical interpretations are incorrect. For instance, there were no statistical association according to your multivariable analysis, yet, your paper keep saying construction sites and water stagnation are strong determinant. This has be modified. Further more, use of P at 0.02 threshold has to be justified with standard epidemiological work.

 Most importantly, since no entomological indices such as larval count, adult house index was measure, the title and the conclusion/recommendation exceed strength of evidence provided. Thus, recommendation should be Precautionary and Perception0informed and not evidence. Also, 'Mosquito density' used in title, tables etc could be modified into something like 'Perceived mosquito density' since this is not measured       

Is the work clearly and accurately presented and does it cite the current literature?

Partly

If applicable, is the statistical analysis and its interpretation appropriate?

Partly

Are all the source data underlying the results available to ensure full reproducibility?

Yes

Is the study design appropriate and is the work technically sound?

Yes

Are the conclusions drawn adequately supported by the results?

No

Are sufficient details of methods and analysis provided to allow replication by others?

Yes

Reviewer Expertise:

Applied Entomology, Medical and Molecular Entomology

I confirm that I have read this submission and believe that I have an appropriate level of expertise to confirm that it is of an acceptable scientific standard, however I have significant reservations, as outlined above.

# Reviewer report: Reviewer response for version 3

**DOI:** 10.5256/f1000research.191677.r436213

**Published:** 2025-12-13

**Authors:** Siraj Khan

**Affiliations:** 1 ICMR-Regional Medical Research Centre, Lahowal, India

The authors have thoroughly addressed all the comments raised in the initial review, providing clear revisions and satisfactory clarifications wherever required. The manuscript has improved substantially in terms of clarity, methodological presentation, and overall scientific quality. All major and minor concerns have been resolved, and no further revisions are necessary at this stage. Therefore, I recommend that the manuscript be accepted for indexing.

Is the work clearly and accurately presented and does it cite the current literature?

Partly

If applicable, is the statistical analysis and its interpretation appropriate?

Partly

Are all the source data underlying the results available to ensure full reproducibility?

Yes

Is the study design appropriate and is the work technically sound?

Yes

Are the conclusions drawn adequately supported by the results?

Partly

Are sufficient details of methods and analysis provided to allow replication by others?

Yes

Reviewer Expertise:

Medical Entomology, Arbovirology, Rickettsial Diseases, Public health

I confirm that I have read this submission and believe that I have an appropriate level of expertise to confirm that it is of an acceptable scientific standard.

# Reviewer report: Reviewer response for version 3

**DOI:** 10.5256/f1000research.191677.r436695

**Published:** 2025-12-11

**Authors:** Asma Rahim

**Affiliations:** 1 Government Medical College, Kozhikode, India

-
  1. Introduction — add a paragraph on coastal urban vector ecology and novelty of Mangalore
  2. Add a conceptual framework ( WHO’s integrated vector management or ecosocial framework)
  3. Split the research questions into Primary and secondary .
  4. Objectives (rewrite, split primary/secondary).Eg:Primary objective
    • To identify environmental and household determinants associated with increased mosquito presence in the urban field practice area of Mangalore.
    Secondary objectives
    • To describe household-level preventive practices and community perceptions towards mosquito-borne diseases.
    • To estimate the prevalence of self-reported mosquito-borne disease in the last 12 months and assess associations with environmental and behavioral factors after adjustment.
  5. Methods — Questionnaire validation (insert a concise methods paragraph).add content validity, pilot test, and Cronbach’s alpha for each construct.
  6. Methods — Operational definitions (insert a short table or list), Mosquito presence (household level, **Nuisance score, **Self-reported mosquito-borne disease, Water stagnation. Eg . Visible standing water that persisted for >48 hours after rain or due to blocked drains in the household compound or immediate neighbourhood.
  7. Sampling : clarify purposive ward selection, add random start for street sampling, acknowledge  exclusion of apartments.
  8. Analysis: Add multivariable logistic regression.
  9. Results: rewrite causal statements, clean tables, add CIs and adjusted ORs.
  10. Tables — formatting checklist
- Each table: give a clear title, state N for the analysis, and column denominators.
- For categorical comparisons, show counts and percentages: “n (%)”.
- For key associations, present crude OR (95% CI) and adjusted OR (95% CI) in a single table.
- Ensure p-values match the test used and report exact values (not only “0.00” or “<0.05”).
- Fix Table numbering and remove “continued” fragments by merging or reformatting long tables.

11. Discussion: add explanation of key findings, strengths and limitations, policy implications.

 12. Limitations: Add an explicit paragraph about seasonality, selection bias, and self-reporting.

Is the work clearly and accurately presented and does it cite the current literature?

Partly

If applicable, is the statistical analysis and its interpretation appropriate?

Partly

Are all the source data underlying the results available to ensure full reproducibility?

Yes

Is the study design appropriate and is the work technically sound?

Partly

Are the conclusions drawn adequately supported by the results?

Partly

Are sufficient details of methods and analysis provided to allow replication by others?

Partly

Reviewer Expertise:

Public health , Community Rheumatology, Health professions education

I confirm that I have read this submission and believe that I have an appropriate level of expertise to confirm that it is of an acceptable scientific standard, however I have significant reservations, as outlined above.

# Reviewer report: Reviewer response for version 2

**DOI:** 10.5256/f1000research.187323.r416581

**Published:** 2025-11-10

**Authors:** Kristan Alexander Schneider

**Affiliations:** 1 University of Applied Sciences Mittweida, Mittweida, Germany

I have reservations against the current version of the article, regarding its format, methodology and content, which I want to lay out below.

**Major comments**

**Format of the article:**

 The result section of the article consists only of tables, without any text describing and explaining the results. The description of the results is mixed into the discussion section. The discussion lacks to critically reflect on limitations, but provides background information, which is better suite for the introduction.

**Abstract:**

 Results: Sometimes percentages are reported as decimal numbers, sometimes spelled out as words. This inconsistency should be avoided, and ideally confidence intervals should be reported too (e.g. exact binomial confidence intervals). When analyzing outcomes of individual questions in the questionnaire, adjustments for multiple testing (e.g. Holm’s correction for FWER) should be applied. To me, it seems neither “water stagnation” nor “construction activity” would have a significant association with mosquito abundance once a correction for multiple testing is applied.

 Conclusions: The conclusions do not reflect the results. If 92% of respondents are aware of mosquito-borne diseases, which is higher than the 91.5% of households that use chemical mosquito prevention, it is hardly justified to claim that there is “a gap in community knowledge and perception of mosquito-borne diseases, even though people are aware of basic precautions,…”.  The conclusions should be aligned with the actual findings.

**Introduction:**

 “7,00,000 deaths” should presumably read “700,000 deaths”. This number needs to be commented. Malaria causes 600,000 to 700,000 deaths per year, whereas other mosquito-borne diseases cause tens of thousands. These numbers should be broken down in more detail.

 The statement “India accounts for 79% of the global malaria burden” cannot be true since 94% of cases and 95% of deaths occur in sub-Sahara Africa. What is true is that India accounts for the majority (around 52%) of malaria cases outside Africa.

 Furthermore, the information on *Anopheles* should be formulated more carefully. Not all *Anopheles* species breed in clean water. The urban vector *Anopheles stephensi*, which becomes invasive in many countries, is known to bead also in unclean water ( *A. stephensi* is prevalent in the study area, cf. Ghosh et al. 2008; this should be mentioned). Moreover, several *Anopheles* species were reported to shift towards more diurnal biting behavior after being targeted by IRS and after deployment of LLITNs.

 I suggest including a map of the study side alongside some of the key climatic characteristics etc. of the region.

 The last sentence of the introduction should be avoided.

**Objectives:**

 Whole sentences should be used in this section.

**Methodology:**

 I do not understand the sample size calculation. It resembles a bit Cochran’s formula but, it has some errors. Before a sample size can be determined, there must be a clear analysis plan. Based on these specifics, the sample size has to be determined.

 The current formula has some problems. If it is Cochran’s formula *Z* *a* *2*, should be *Z ^2^*, with *Z*=1.96 being the 97.5% quantile of the standard normal distribution rather than the 95% confidence interval. Second, *d2* should be *d ^2^*, with *d* being the margin of error, rather than the relative precision (whatever this should be).  In any case it is unclear how the authors arrive at a sample size of *N=95*. With Cochran’s formula and 20% non-responders, I calculated *N=68*.

 In the second paragraph on page 6 the methodology of the questionnaire cannot be assessed. The sentence “A pretested internally validated physical questionnaire designed after extensive literature review…” dose not sufficiently describe the methodology. A description how the literature research was performed is necessary. It should also explain in detail how the questionnaire was validated and by whom. It should be more prominently stated that the is provided as supplement. I am also wondering whether the questionnaire asked about insecticide resistance. This is an issue in the area given that *A. stephensi* is prevalent there.

**Results:**

 The result section lacks any main text. Rather tables are listed without any description. In general, it is good practice to provide confidence intervals alongside the frequency estimates.

 Regarding the information in Table 1, 5.2% of the participants are illiterate. I am wondering how these participants completed the questionnaire. This needs to be described in the methods.

 I find the questions and answers in Table 2 rather unfortunate. Presumable because not the actual question is provided. For instance, “Presence of mosquitoes inside the house” sounds like a dichotomous question to me, or like a quantitative question, however the answers “Mild”, and “Moderate to Severe” are rather qualitative. It is also unclear from the questionnaire what exactly was reported and which questions and how they were aggregated. “Time of the day when mosquitoes mostly bite” has answers “Morning”, “Evening”, and “Night”.  These answers are unfortunate, since there is no clear-cut definition for “Morning”, “Evening”, and “Night”. To some 10pm might be evening, to others it would be night. Moreover, this might not have anything to do with the actual biting behavior, because it is not uncommon to not notice when mosquitoes are biting. If you wake up with a couple of mosquito bites, it is unclear when they bite. I also found the possible answers “Mild” and “Moderate to Severe” to the question “Overall mosquito nuisance in locality compared to past one year” not ideal. What is if mosquito nuisance is significantly reduced in comparison to the previous years. “Mild” is not a good qualitative summary of significantly less.

 In Table 7, I am wondering about the mosquito born diseases.  Apparently, of the 28 persons, who suffered from a mosquito borne disease, 71.4% had dengue. While dengue incidence is 2.5 times higher than malaria in the region, this seems to fit in the picture. However, in this context it is important to note that several serotypes circulate in this area (DENV-2, DENV-3, and also DENV-1). Since one acquires lasting immunity against the first infecting serotype (which typically cause mild infections with unspecific symptoms) and temporal immunity against the others, one can make the implication that the persons with a dengue infection had a severe infection. Otherwise, dengue would not be diagnosed. It is important to discuss these facts.

**Discussion:**

 The first two lines make little sense given the study design. The study was conducted in an urban setting close to the Medical College. Hence, it is not surprising that study participants report good access to healthcare.  Moreover, the access to healthcare is less important than the ability to properly diagnose vector-borne diseases. Furthermore, the study had a focus on mosquitoes, not on healthcare access.

 The rest of the first paragraph also makes no sense. The study in Florida is concerned with a totally different mosquito population. I do not see the connection between residents in Florida feeling bothered by mosquitoes and a population in India.

 The compassion between India and Mozambique in the second paragraph also is of limited relevance. Also, the relevance of the fourth and fifth paragraph is unclear to me.

 The third paragraph states that 50% of the mosquitoes were often found inside the house. I cannot find this result in any of the Tables.

 The first paragraph on page 13 should discuss dengue in more detail.

**Conclusions:**

 It is stated that the study adopts a “novel,” integrated approach. To me, it is unclear what is novel about a questionnaire?

 The term “correlation” is not correctly used. Correlations between metric (or ordinal) variables. Otherwise, it is an association.

 In general, I miss a clear discussion on the limitations of the study. I also think spatial repellants or larvicides should be discussed somewhere in the text.

**Minor comments**

 Abstract:

1. I suggest using whole sentences under the heading “Objectives”.
2. Be specific about the study area and explain what the “Department of Community Medicine” is.
3. Results: “… as mosquito preventive measure.” -> “… for mosquito prevention.”

Introduction: When you mention dengue and chikungunya, you might also mention West Nile virus.

Is the work clearly and accurately presented and does it cite the current literature?

No

If applicable, is the statistical analysis and its interpretation appropriate?

Partly

Are all the source data underlying the results available to ensure full reproducibility?

Yes

Is the study design appropriate and is the work technically sound?

Partly

Are the conclusions drawn adequately supported by the results?

No

Are sufficient details of methods and analysis provided to allow replication by others?

No

Reviewer Expertise:

Global health, mathematical modeling, biostatistics, bioinformatics

I confirm that I have read this submission and believe that I have an appropriate level of expertise to state that I do not consider it to be of an acceptable scientific standard, for reasons outlined above.

## References

[1] : Observations on sporozoite detection in naturally infected sibling species of the Anopheles culicifacies complex and variant of Anopheles stephensi in India. *Journal of Biosciences* .2008;33(3) : 10.1007/s12038-008-0052-5 333-336 10.1007/s12038-008-0052-5 19005232

# Response

**Published:** 2025-11-17

**Authors:** DR JITHIN SURENDRAN

**Affiliations:** COMMUNITY MEDICINE, KMC Mangalore, MANGALORE, KARNATAKA, India

We deeply appreciate the reviewer’s comprehensive and constructive comments. We have carefully addressed each point, and the manuscript has improved substantially as a result.

 Point-by-Point Response to Reviewer Comments

 Major Comments

 1. Format of the article (Results section & Discussion)

**Response: **A full narrative description has now been incorporated into the Results section. This text had been included in the earlier revised version of the manuscript but was unfortunately not visible to the reviewer during the previous round of assessment. All misplaced results were removed from the Discussion and relocated appropriately. Background content has been shifted to the Introduction. A detailed Limitations subsection has been added.

 2. Abstract 

**Response: **Percentages are now presented consistently in numerical form. Exact binomial confidence intervals were added. Holm’s correction was applied for multiple comparisons, and results were updated—“water stagnation” and “construction activity” no longer show significant association. The Conclusions section has been fully revised to accurately reflect the study findings without overstating knowledge gaps.

 3. Introduction 

**Response: **Global mortality figures were corrected and broken down. The statement on India’s contribution to global malaria burden was corrected to indicate its burden outside Africa.

 4. Introduction – Anopheles ecology inaccuracies

**Response: **Statements on Anopheles breeding habits were corrected. The role of *A. stephensi,* its prevalence locally, ability to breed in unclean water, and its shifting biting patterns have been incorporated with updated references (including Ghosh et al. 2008).

 5. Study area map suggested

**Response: **A geographical map of the study area has been added as a figure, along with a description of local climate and demographics.

 6. Last line of introduction should be avoided

**Response: **The final sentence of the introduction has been deleted.

 7. Objectives not in complete sentences

**Response: **Objectives have been rewritten into full, clear sentences.

 8. Methodology – unclear sample size calculation

**Response: **The sample size section has been rewritten using correct Cochran’s formula notation (Z² and d²). Assumptions are now clearly stated, and the revised calculation aligns with response rate considerations.

 9. Methodology – insufficient description of questionnaire development and validation

**Response: **A detailed explanation of questionnaire development—including literature review, item generation, pretesting, and validation—has been added. Content validation by three public health specialists and two medico-social workers was performed using a four-point Likert scale to derive I-CVI and S-CVI/Ave values. Items with I-CVI < 0.78 were revised or removed, and an S-CVI/Ave ≥ 0.8 confirmed good validity; the full questionnaire is now included as a supplementary file.”

 10. Methodology – question about insecticide resistance

**Response: **Questions were not included on insecticide resistance; this has now been explicitly stated under methodological limitations.

 11. Results section lacked narrative and CIs

**Response: ** Comprehensive narrative text now accompanies each table. Exact binomial confidence intervals were added to all proportion estimates.

 12. Table 1 – handling of illiterate participants unclear

**Response: **Methods now explain that trained field investigators were administered the questionnaire orally to illiterate participants.

 13. Table 2 – issues with response options, clarity, and interpretability

**Response: **Table 2 has been revised for clarity. Question wording and response scales have been standardized, and explanatory notes added. Ambiguous qualitative categories have been justified in methodology and clarification on the categorization of mosquito biting times has been added directly within the respective table.

 14. Table 7 – dengue proportion requires context of serotypes

**Response: **Discussion now includes an explanation of dengue serotype circulation (DENV-1, 2, 3), immunity patterns, and why diagnosed cases likely reflect moderate–severe infections.

 15. Discussion – irrelevant comparisons and unclear statements

**Response: **Paragraphs on healthcare access, Florida mosquito study, and Mozambique comparison have been removed and rewritten for relevance respectively. Statements unsupported by results were removed.

 16. Missing statement: 50% mosquitoes found inside homes

**Response: **This statement was removed as it was not supported by data.

 17. Dengue discussion insufficient

**Response: **The dengue section has been expanded to include serotype diversity, severe disease likelihood, and regional epidemiology.

 18. Conclusions – novelty claim incorrect; misuse of “correlation”

**Response: **Claims of novelty have been removed, and the conclusions now focus on the integrated approach used as study objectives. The term “association” is now used appropriately throughout.

 19. Missing Limitations & omission of spatial repellents/larvicides

**Response: **A detailed Limitations section has been added, including the absence of entomological indices and resistance testing. Discussion now includes spatial repellents and larvicidal interventions.

 Minor Comments

 1. Objectives should use full sentences

**Response: **Revised accordingly.

 2. Abstract: specify study area and “Department of Community Medicine”

**Response: **The abstract now specifies the study area and explains the institutional context.

 3. Abstract: “mosquito preventive measure” wording

**Response: **Corrected to “for mosquito prevention.”

 4. Introduction: mention West Nile virus

**Response: **West Nile virus has been included in the list of mosquito-borne diseases.

# Reviewer report: Reviewer response for version 1

**DOI:** 10.5256/f1000research.181258.r398023

**Published:** 2025-08-22

**Authors:** Dr Divya Bharathi

**Affiliations:** 1 Kempegowda Institute of Medical Sciences, banglore, India

Title -  informative and specific, can include the place of the study.

 Introduction- Para 2, Line - 2 the arthropods to these arthropods, should be corrected.

 Avoid redundancy (e.g., restating that mosquitoes transmit diseases multiple times).

 Review of literature- relevant studies are incorporated. 

 Methodology- Mention if multiple data collectors used and operational definitions for "moderate to severe mosquito nuisance"

 Discussion- Does not discuss possible confounders

 Conclusion- ****Well-summarized. Recognizes the knowledge–practice gap.

 Overall the study is Context-specific, regionally relevant.Combines environmental, behavioral, and health outcome data.

Is the work clearly and accurately presented and does it cite the current literature?

Yes

If applicable, is the statistical analysis and its interpretation appropriate?

Yes

Are all the source data underlying the results available to ensure full reproducibility?

Partly

Is the study design appropriate and is the work technically sound?

Yes

Are the conclusions drawn adequately supported by the results?

Yes

Are sufficient details of methods and analysis provided to allow replication by others?

Yes

Reviewer Expertise:

Communicable diseases, Rabies, Vector borne diseases, MCH

I confirm that I have read this submission and believe that I have an appropriate level of expertise to confirm that it is of an acceptable scientific standard.

# Response

**Published:** 2025-08-26

**Authors:** DR JITHIN SURENDRAN

**Affiliations:** COMMUNITY MEDICINE, KMC Mangalore, MANGALORE, KARNATAKA, India

Thank you very much for reviewing the revised manuscript and confirming your approval. I sincerely appreciate your careful reading, thoughtful feedback, and guidance, which have greatly contributed to improving the clarity, rigor, and overall quality of the article. .

 The manuscript has been further revised to address reviewer comments: in the Introduction, typographical errors (e.g., “the arthropods” → “to these arthropods”) have been corrected, and redundancy regarding mosquito-borne diseases has been removed. Relevant studies have been incorporated in the merged Review of Literature. The Discussion has been refined to acknowledge possible confounders, and the Conclusion highlights the knowledge–practice gap.

 Overall, the study remains context-specific and regionally relevant, integrating environmental, behavioral, and health outcome data to provide actionable insights. Your confirmation reassures us that the manuscript accurately reflects the study’s objectives and findings.

 Thanking you

 With regards

 Corresponding author

# Reviewer report: Reviewer response for version 1

**DOI:** 10.5256/f1000research.181258.r400061

**Published:** 2025-08-13

**Authors:** Siraj Khan

**Affiliations:** 1 ICMR-Regional Medical Research Centre, Lahowal, India

**Summary**

 The article entitled ‘ **Determinants of urban mosquito population density and community responses: A cross-sectional study’** is well written and emphasizes the importance of community-based interventions and identifies gaps in local authority involvement, which can influence municipal policy. While the study is relevant and well-organized, it requires stronger justification of its novelty, broader statistical analysis, and clearer resolution of paradoxical findings regarding preventive practices. Addressing these would significantly improve the rigor and clarity of the manuscript.

**1. Is the work clearly and accurately presented and does it cite the current literature?**

 a. ‘An initial survey conducted in Mangalore taluk identified 26 mosquito species across six genera’- add reference here.

 b. Several references are cited without corresponding numbers in the bibliography (e.g., references 14–21). Ensure accurate and complete citation.

**2.If applicable, is the statistical analysis and its interpretation appropriate?**

 a. The sample size (n=95) is small for making generalizable statements, especially in a city with high heterogeneity in socioeconomic and environmental factors.

 b. The statistical data will be more reliable when it is presented confidence intervals in addition to p-values.

 c. The households using coils and repellents had higher disease prevalence is discussed but not thoroughly explained. Could reverse causation or confounding (e.g., higher-density mosquito areas using more coils) be responsible?

 d. No multivariate analysis e.g., logistic regression was performed, which could have clarified confounding between overlapping risk factors like vegetation, construction, and socioeconomic variables.

**3. Are the conclusions drawn adequately supported by the results?**

 a. While the problem is important, similar studies have been published in other endemic settings. Authors are advised to clearly express the novelty—what new insights does this paper provide that prior research (e.g., Wilke et al., 2020; Garbin et al., 2021) did not.

 b. The graphical representation of data is more understandable as demographic data is concerned.

**Other comments**

 a **. **Scientific names like *Aedes aegypti* and *Anopheles* should be consistently italicized.

 'b. Aedes aegypti and Aedes albopictus are mosquitoes that belong to the Culicines category,’ -what does ‘category’ means here?

 c. Review of literature section can be merged with Introduction section.

Is the work clearly and accurately presented and does it cite the current literature?

Partly

If applicable, is the statistical analysis and its interpretation appropriate?

Partly

Are all the source data underlying the results available to ensure full reproducibility?

Yes

Is the study design appropriate and is the work technically sound?

Yes

Are the conclusions drawn adequately supported by the results?

Partly

Are sufficient details of methods and analysis provided to allow replication by others?

Yes

Reviewer Expertise:

Medical Entomology, Vector borne Diseases, Rickettsial diseases, Public health research

I confirm that I have read this submission and believe that I have an appropriate level of expertise to state that I do not consider it to be of an acceptable scientific standard, for reasons outlined above.

# Response

**Published:** 2025-08-26

**Authors:** DR JITHIN SURENDRAN

**Affiliations:** COMMUNITY MEDICINE, KMC Mangalore, MANGALORE, KARNATAKA, India

We would like to express our gratitude to the reviewer for the insightful and constructive feedback on our manuscript. The comments have been invaluable in refining the manuscript, and we have addressed each suggestion thoroughly in the responses below.

 1 a. " *An initial survey conducted in Mangalore taluk identified 26 mosquito species across six genera*"- The reference has been added as suggested.

 1.b. Several references are cited without corresponding numbers in the bibliography- All references have been thoroughly verified to ensure that the citations are accurate and complete.

 2 a. The sample size (n=95) is small for making generalizable statements, especially in a city with high heterogeneity in socioeconomic and environmental factors- We ensured representative coverage across wards by dividing the households equally among the selected 5 wards (19 households per ward) out of 60 wards. The sampling strategy has been mentioned in methodology. Hope, this systematic approach  maximize representativeness and reduce selection bias within the constraints of the study.

 2.b. The statistical data will be more reliable when it is presented confidence intervals in addition to p-values- The table 7 & 8 have been revised to present odds ratios with 95% confidence intervals alongside the previously reported p-values.

2.c The households using coils and repellents had higher disease prevalence is discussed but not thoroughly explained. Could reverse causation or confounding (e.g., higher-density mosquito areas using more coils) be responsible?- The justification has been provided in the last paragraph of discussion section.

 2.d. No multivariate analysis e.g., logistic regression was performed, which could have clarified confounding between overlapping risk factors like vegetation, construction, and socioeconomic variables- The table 7 & 8 have been revised incorporating logistic regression analysis.

 3.a. While the problem is important, similar studies have been published in other endemic settings. Authors are advised to clearly express the novelty—what new insights does this paper provide that prior research (e.g., Wilke et al., 2020; Garbin et al., 2021) did not- The study adopts novel objectives, focusing on an integrated approach that identifies key determinants of mosquito presence while simultaneously evaluating community measures, perceptions, and self-reported disease burden, providing context-specific insights from Mangalore that extend beyond previous studies in other settings. This has been incorporated into the conclusion.

 3.b. The graphical representation of data is more understandable as demographic data is concerned- We appreciate the suggestion regarding graphical representation. However, we have retained the tabular format for demographic data, as it allow us for clear presentation of detailed counts and percentages across multiple categories, which may be less effectively conveyed in graphs.

 a **. **Scientific names like *Aedes aegypti* and *Anopheles* should be consistently italicized- This has been incorporated across the article.

 b. Aedes aegypti and Aedes albopictus are mosquitoes that belong to the Culicines category,’ -what does ‘category’ means here?- This has been revised in introduction section Para 3, line 1.

 c. Review of literature section can be merged with Introduction section- The Review of Literature section has been merged with the Introduction

 We sincerely thank the reviewer for their valuable time, insightful comments, and constructive suggestions, which have greatly helped us improve the clarity, rigor, and overall quality of our manuscript. We hope that the revisions adequately address all concerns, and we look forward to the reviewer’s further feedback.
